# Optimizing sparse and skew hashing: faster *k*-mer dictionaries

**DOI:** 10.1093/bioinformatics/btag264

**Published:** 2026-07-07

**Authors:** Giulio Ermanno Pibiri, Rob Patro

**Affiliations:** DAIS, Ca’ Foscari University of Venice, Venice, Italy; Department of Computer Science, University of Maryland, College Park, MD 20440, United States

## Abstract

**Motivation:**

Representing a set of *k*-mers—strings of length *k*—in small space under fast lookup queries is a fundamental requirement for several applications in Bioinformatics. A data structure based on *sparse and skew hashing* (SSHash) was recently proposed for this purpose (Pibiri 2022): it combines good space effectiveness with fast lookup and streaming queries. It is also *order-preserving*, i.e. consecutive *k*-mers (sharing a prefix-suffix overlap of length k−1) are assigned consecutive hash codes which helps compressing satellite data typically associated with *k*-mers, like abundances and color sets in colored De Bruijn graphs.

**Results:**

We study the problem of accelerating queries under the sparse and skew hashing indexing paradigm, without compromising its space effectiveness. We propose a refined data structure with less complex lookups and fewer cache misses. We give a simpler and faster algorithm for streaming lookup queries. The refined architecture translates to substantial performance gains, outperforming the original version of SSHash in both index construction speed and query efficiency. Compared to indexes with similar capabilities and based on the Burrows-Wheeler transform, like SBWT and FMSI, SSHash is significantly faster to build and query. SSHash is competitive in space with the fast (and default) modality of SBWT when both *k*-mer strands are indexed. While larger than FMSI, it is also more than one order of magnitude faster to query.

**Availability and Implementation:**

The SSHash software is available at https://github.com/jermp/sshash, and also distributed via Bioconda. A benchmark of data structures for *k*-mer sets is available at https://github.com/jermp/kmer_sets_benchmark. The datasets used in this article are described and available at https://zenodo.org/records/17582116.

## 1 Introduction

Efficient representation and indexing of large sets of *k*-mers (substrings of fixed length *k* appearing in longer strings) is a central primitive in modern Bioinformatics. To tackle this problem, Pibiri (2022) recently introduced a data structure based on *sparse and skew hashing* (SSHash, hereafter). Apart from being compact and fast to query, its distinctive feature is that it is *order-preserving: k*-mers that overlap by k−1 characters are likely assigned *consecutive* hash codes. This property makes it particularly well suited for compressing satellite data associated with *k*-mers, such as abundances and color sets in colored De Bruijn graphs, because values referring to consecutive *k*-mers tend to be very similar (if not identical), and storing them consecutively significantly improves compression, as well as, locality of reference. Indeed, state-of-the-art indexes for colored (Campanelli *et al.* 2024, Fan *et al.* 2024) and positional (Fan *et al.* 2023) De Bruijn graphs rely on SSHash for this reason. Improving it thus directly impacts on the performance of such indexes.

In this work, we revisit the sparse and skew hashing paradigm with the goal of improving query performance without compromising space effectiveness. We introduce a range of refinements, including: (i) a less complex query logic, (ii) a new layout that reduces the number of cache misses per query and leads to consistently better runtime in practice, (iii) a simpler and faster algorithm for *streaming* lookup queries. This is a query modality where lookup queries are issued for consecutive *k*-mers coming from reads or longer sequences. This modality is the one typically used in practice, e.g. to perform pseudo alignment on colored De Bruijn graphs (Bingmann *et al.* 2019, Alanko *et al.* 2023b, Fan *et al.* 2024).

We experimentally evaluate the new SSHash design (which outperforms the previous one in both query and construction time) against the latest state-of-the-art *k*-mer dictionaries that offer similar functionalities, such as SBWT (Alanko *et al.* 2023a) and FMSI (Sladký *et al.* 2026), both based on the Burrows and Wheeler (1994) transform (BWT). Our results indicate that SSHash achieves significantly better query performance and faster construction times. In terms of space usage, SSHash is competitive with SBWT in the bidirectional model (i.e. when both strands of each *k*-mer are indexed). This is the *de-facto* model used by applications built atop these indexes. The space usage of SSHash is actually better for larger *k*. It remains, on the other hand, more space-consuming than FMSI, but more than an order-of-magnitude faster to query.

## 2 Preliminaries

In this section we fix notation and illustrate the basic tools we use throughout the paper to solve the following problem.

Problem 1 (The *k*-mer dictionary problem).

Given a collection X of strings and an integer k>0, build a data structure that represents all n distinct k-mers of X so that the following queries are efficient:

- For any k-mer x, LOOKUP(x) returns a handle h∈[1..U] (where U≥n) if x appears in X, or ⊥ otherwise. For any x≠y, LOOKUP(x)≠LOOKUP(y) must hold.
- For any h∈[1..U], Access(h) retrieves the k-mer x if LOOKUP(x)=h, or returns the empty string ε otherwise.
- For any string s of length at least k, LOOKUP(s) answers LOOKUP(x) for all k-mers x of s.

**Basic notation.** Given a string *s*, we indicate with |s| its length and with s[i..j) its substring of length j−i beginning with the symbol in position *i* and terminating with that in position j−1, for any 1≤i<j≤|s|+1. (We use 1-based indexing throughout the paper.) Notation “x∈s” means that *x* appears as a substring of *s* and we let pos(x,s) be the start position of *x* in *s*.

In this paper we consider strings over the DNA alphabet {A,C,G,T}. Each symbol in this alphabet has a complement: the complement of A is T (and vice versa), and the complement of C is G (and vice versa). The *reverse complement* of the string *s*, indicated with $\bar{s}$, is the string where all the characters of *s* appear in reverse order and complemented. Since $\bar{s}$ can be obtained from *s* (and vice versa), we consider them identical.

The *k*-mer *spectrum* of the string *s*, ${\text{spect}}_{k}(s)$, is the set of *k*-mers of *s*. Similarly, we define ${\text{spect}}_{k}(X)=\cup_{s\in X}{\text{spect}}_{k}(s)$. A *spectrum-preserving string set* (or SPSS) for X is another set of strings $S=\{s_{i}\}$ such that $|s_{i}|\geq k$ for each $s_{i}$ and ${\text{spect}}_{k}(S)={\text{spect}}_{k}(X)$. In this work we only consider SPSSs that do *not* repeat *k*-mers, that is ${\text{spect}}_{k}(s_{i})\cap {\text{spect}}_{k}(s_{j})=\emptyset$ for any $s_{i},s_{j}\in S$, i≠j. For the rest of the paper, let $S=\{s_{i}\}$ be an SPSS for X with $N=\sum_{i}|s_{i}|$ (An algorithm that computes S such that both |S| and *N* are minimum is known (Schmidt and Alanko 2023), improving over previous heuristics (Rahman and Medvedev 2020, Břinda *et al.* 2021). In this case, the strings in S are called *eulertigs*. This is the form of S we are going to use in the experimental analysis in Section 8. Furthermore, we note that if duplicate *k*-mers are allowed in S, the SPSS of minimum total length is composed of *matchtigs* and that SSHash can index matchtigs as well (Schmidt *et al.* 2023).). Given a *k*-mer *x* of X, it follows that $\exists !s_{i}\in S$ such that either $x\in s_{i}$ or $\bar{x}\in s_{i}$. Intuitively, S provides a natural basis for a data structure solving Problem 1 because it contains *n* distinct *k*-mers from the input X, without repetitions.

We call *S[*1.*N]* the string obtained by concatenating all the strings $s_{i}$ in some fixed order, and indicate via $p_{i}$, the position at which $s_{i}$ begins in *S*. Thus, $s_{i}=S[p_{i}..p_{i+1})$ for i=1..|S| and $p_{|S|+1}=N+1$. Let $P=\{p_{i}\}$ in the following.

**Minimizers and super-*k*-mers.** A minimizer sampling scheme is defined by a triple (m,k,O), where m,k∈N, m<k, and O is an order over all *m*-long strings. Given a *k*-mer *x*, the minimizer μ of *x* is the leftmost *m*-mer of *x* such that O(μ)≤O(y) for any other *m*-mer *y* of *x*. To simplify notation, we indicate the minimizer of the *k*-mer *x* with MINI(x) in the following, without specifying the parameters *m* and O. In practice, the order O is usually random (e.g. implemented as a pseudo-random hash function).

A string is said to be *sampled* at the positions of the minimizers of its *k*-mers. For a string composed of *N* i.i.d. random characters, and when *m* is sufficiently long, the expected number of *distinct* sampled positions is approximately 2/(k−m+2)·(N−m+1) [see Theorem 3 by Zheng *et al.* (2020) for details]. In other words, random minimizers are *sparse* along the string; in expectation, the start positions of two consecutive minimizers are (k−m+2)/2 characters apart.

We give two more definitions. For any *k*-mer *x*, we define its *canonical minimizer* as $\text{CMini}(x):=\min\{M\text{INI}(x),M\text{INI}(\bar{x})\}.$

A *super-k-mer* of a string *s* is a maximal substring of *s* where a given property R(x) does not change for all *k*-mers x∈s. We say that *s* is *parsed* into super-*k*-mers by property *R*. As an example, consider the string s=TCAAGTTGGCCT…, and let k=7. Let R(x) be the lexicographic minimizer of length m=3. Then the first super-*k*-mer of the string would be TCAAGTTGG because its three *k*-mers (TCAAGTT, CAAGTTG, and AAGTTGG) all have minimizer AAG according to the lexicographic order of *m*-mers. Note that this substring is maximal because the fourth *k*-mer of *s* does not have AAG as minimizer.

**Model of computation: cache misses.** To analyze the time complexity of an algorithm, we count the number of cache misses that it spends, as practical performance critically relates to them. We use the following simple model: C(Q):=⌈Q/B⌉ is the number of cache misses that occur when the algorithm reads *Q consecutive* bits, using a cache with page (or *line*) size equal to *B* bits (Vitter 2001). For most architectures the value *B* is 512 (64 bytes). That is, *B* spans several memory words. We assume that $\left\lceil {\;\text{log}\;}_{2}(N)\right\rceil \leq B$ for the rest of the paper (We omit ceiling operators when they are not essential.).

**Minimal perfect hash functions.** A function f:X→{1,…,|X|} is said to be a minimal perfect hash function for the set *X* if f(x)≠f(y) for all x,y∈X, x≠y. In other words, a MPHF for *X* maps its keys into the first |X| natural numbers without collisions. Note that f is *not* defined for a key not inX, thus permitting to implement f with just a constant amount of space per key. Indeed the theoretical space lower bound for representing a MPHF is (less than) ${\;\text{log}\;}_{2}(e)\approx 1.443$ bits per key (Mehlhorn 1982), assuming the keys of *X* are drawn from a large universe and |X| is large as well.

Many practical constructions for MPHFs are known that scale well to large datasets, take little space on top of the lower bound, and retain very fast evaluation time. See the recent survey by Lehmann *et al.* (2026) for an overview of various techniques. In this paper, we use techniques based on *bucket placement* that specialize in very fast evaluation time, requiring (under proper tuning) 1 cache miss per evaluation (Pibiri and Trani 2021).

**Elias-Fano sequences.** Consider a sorted sequence *A* of *n* integers 0≤A[1]<A[2]<⋯<A[n]<U for some universe size *U*. The query Successor(x) returns the smallest integer y∈A that is y≥x (assuming, w.l.o.g., x≤A[n], so that the result is well-defined). We use the following result, based on Elias-Fano codes (and point the interested reader to the Supplementary Material for details).

Theorem 1 (Elias-Fano).

There exists a representation of A that takes at most EF(n,U)=n(ℓ+3) bits and:

1. For o(n) extra bits, access to the i-th element, can be supported with, at most, $1+C_{\text{sel}}$ cache misses.
2. For o(n) extra bits, Successor can be supported with, at most, $C_{\text{sel}}+C_{\text{scan}}$ cache misses.
3. For O(n log n) extra bits, Successor can be supported with, at most, $1+C_{\text{scan}}$ cache misses.

The quantities $C_{\text{sel}}$ and $C_{\text{scan}}$ are $C_{\text{sel}}=2+C(O({\;\text{log}\;}^{4}n))$ and $C_{\text{scan}}=C(U/n)+C(\ell \cdot (U/n+1))+C(\Delta_{A})$, where $\Delta_{A}:=\underset{1\leq i<n}{\max}\{\left\lfloor \frac{A[i+1]}{2^{\ell }}\right\rfloor -\left\lfloor \frac{A[i]}{2^{\ell }}\right\rfloor \}$, and $\ell =\left\lfloor {\;\text{log}\;}_{2}(U/n)\right\rfloor$.

## 3 A general indexing framework

With the background and notation fixed in Section 2, the goal of this section is to define a universal, abstract framework that captures the fundamental architecture of any hash-based *k*-mer index for S. This framework comprises four modular components. By isolating these core components, we can systematically derive and compare both existing tools and our own approach, as different time/space trade-offs can be obtained depending on the choice of each module. While the terminology here is necessarily abstract to maintain generality, we will ground these concepts with concrete examples from the literature.

Definition. The four components of the framework are:

1. A *k*-mer transformation function Φ. This function takes a *k*-mer *x* as input and computes a value ϕ=Φ(x).
2. A table *L[*1..*M]*, for some M≤n.
3. A function *f* mapping ϕ to an integer t∈[1..M].
4. The string *S[*1..*N]* paired with the start positions $P=\{p_{i}\}$.

The interaction between these components is described by


L[t]=loc(ϕ), t=f(ϕ), and ϕ=Φ(x)

where loc(ϕ) is the *locate set* of ϕ, a sorted set of integers for which the following property holds.

**Property 1**.

For each j∈loc(ϕ), there exists a string $s_{i}=S[p_{i}..p_{i+1})$ and a k-mer $x\in s_{i}$ such that Φ(x)=ϕ and $\text{pos}(x,S)\in d(j)\subseteq [p_{i}..p_{i+1}-k]$.

The function d(j) is a *displacement* (or *decoding*) function that maps the integer *j* to a set of *k*-mer positions. (We will add more parameters to the function whenever necessary.)

**Intuition.** This framework abstracts the query pipeline of a hash-based *k*-mer index. To achieve space savings, the framework first applies a transformation Φ (e.g. computing a minimizer) to a given *k*-mer *x*, deriving a representative signature ϕ. Because the set of these signatures is typically much smaller than the set of all unique input *k*-mers, the overall memory footprint is reduced. The function *f* then maps this signature to a specific entry in the table *50*—the core data structure alongside the string *S*. To further avoid the memory bottleneck of storing absolute text coordinates for every individual *k*-mer, the index instead stores a shared locate set loc(ϕ) containing encoded identifiers *j*. Finally, the displacement function *d* decodes these identifiers to recover the exact coordinates of *x* in *S*.

**Algorithm 1** The Lookup algorithm for a *k*-mer *x*, using the framework illustrated in Section 3.

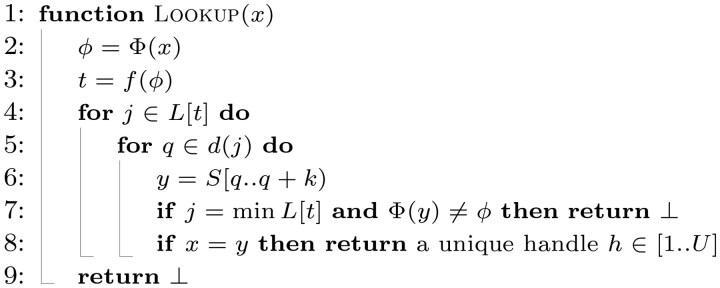

**Queries.** Algorithm 1 concretely shows how LOOKUP(x) is implemented following this framework. Again, the idea is to use the value ϕ=Φ(x) to retrieve the set loc(ϕ) and use it to locate the *k*-mer *x* in *S*. Note that all *k*-mers *y* read at Line 6 must be such that Φ(y)=ϕ to be present in the input because all the elements j∈L[t] satisfy Property 1. If, instead, Φ(y)≠ϕ, it means that the value ϕ was not observed for any *k*-mer in the input, hence the search can be terminated directly.

The algorithm for Access(h), instead, depends on the choice of *h* at Line 8 of Algorithm 1. For example, if the choice h=q is made, then U=N−k≥n (i.e. Lookup does not map *k*-mers into the minimal range *[*1.*n]*, unless |S|=1) and Access(h) is trivial as the *k*-mer S[h..h+k) is returned directly. Instead, if we opt for h=q−(i−1)·(k−1) where *i* is such that $p_{i}\leq q\leq p_{i+1}-k$, then we make the mapping minimal, i.e. h∈[1..n]. In this case, the value *i* can be computed using the query Successor(q) over *P* (for example, representing *P* with the data structure from Theorem 1). Upon Access, however, one has to compute *i* from *h* and the sequence *P* to derive *q*, and lastly retrieve S[q..q+k).

As the number of cache misses of Algorithm 1 depends on specific choices of data structures for the framework, we defer this analysis to subsequent sections.

**Examples.** So far in our description, components such as the locate table *50*, as well as the definitions of Φ, *f*, and loc(ϕ), are treated abstractly to maintain generality. However, the framework is robust enough to model a wide variety of designs; in the examples below, we provide concrete instantiations of these structures, ranging from simple hash tables to tools like Pufferfish, BLight, and SSHash.

A naïve solution would be to let Φ be a (pseudo) random hash function. Then loc(ϕ) would contain the positions *j* in *S* of all the *k*-mers *x* such that ϕ=Φ(x). The function *f* would map the signature ϕ within the bounds of the table *50*, e.g. f(ϕ)=ϕmodM; the displacement function would just be the identity, i.e. d(j)={j}. The key issue of this solution is, obviously, its space usage. First, *L* stores a position for each *k*-mer in the input, thus spending ${\;\text{log}\;}_{2}(N)$ bits per *k*-mer; second, the number of entries of *L* (i.e. *M*) should be chosen large enough, say M=Θ(n), to achieve fast lookup times.

This issue can be mitigated by letting *f* be a MPHF for the set of hash codes {Φ(x)} (assuming these are all distinct). That is, {Φ(x)} is mapped bijectively onto the minimal range *[*1.*n]* for *n* input *k*-mers, so that M=n and loc(ϕ) contains the unique position in *S* of the *k*-mer *x* such that ϕ=Φ(x). To save even more space, loc(ϕ) can store the largest multiple of a chosen quantum v>1 that is smaller than (or equal to) the position of *x*, effectively spending ${\;\text{log}\;}_{2}(N/v)$ bits per *k*-mer instead of ${\;\text{log}\;}_{2}(N)$ bits. The displacement function *d* would then indicate the suitable range (of length *v*, at most). This solution is adopted by the Pufferfish index (Almodaresi *et al.* 2018).

Minimizers can be used to improve space usage by letting Φ=MINI and the strings in S be parsed into super-*k*-mers by Φ. The super-*k*-mers can then be grouped by minimizer, i.e. all super-*k*-mers having the same minimizer are concatenated in the string *S*. Grouping by minimizer naturally leads to a collection of MPHFs, one function per group, to implement the mapping function *f*. Each MPHF now maps a *k*-mer appearing in the super-*k*-mers of the group to its *relative* position in the group. As positions are relative to a group, the space usage improves compared to that of Pufferfish. This is the solution implemented in the BLight index (Marchet *et al.* 2021).

## 4 Sparse and skew hashing

We review and analyze the sparse and skew hashing scheme (SSHash) (Pibiri 2022) in this section, as it lays the foundation for the new development in subsequent sections. Also, we explain how SSHash can be described as a specific instance of the framework from Section 3.

**Figure 1:**
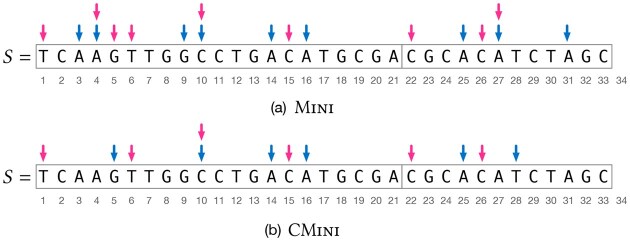
An example string *S* of length N=33 resulting from the concatenation of two strings whose start positions in *S* are $p_{1}=1$ and $p_{2}=22$. There are 21 *k*-mers for k=7 in the string. The blue arrows indicate the start positions of random minimizers of length m=3, while the red arrows indicate the start positions of the corresponding super-*k*-mers. In (a), *S* is parsed into super-*k*-mers by Mini; in (b), by CMini. The lexicographic order of *m*-mers is used to compute minimizers.

**Sparse hashing.** The string *S* is parsed into super-*k*-mers by Φ, where Φ is either Mini or CMini, and each j∈loc(ϕ) is the start position of a super-*k*-mer having minimizer ϕ (red arrows in the example of Fig. 1 at page 1). Note that this saves space compared to BLight (Marchet *et al.* 2021) because the trailing k−1 characters of each super-*k*-mer are encoded once (i.e. the super-*k*-mers are left where they appear in *S*).

Let $M=\{\Phi (x)|x\in {\text{spect}}_{k}(S)\}$. The function *f* is a MPHF for M, hence M=|M|. This choice of *f* also produces a space saving because the MPHF is built over the minimizers rather than the *k*-mers. As minimizers are *sparse* in the string *S*, the cost of the MPHF is small (e.g. less than 0.5 bits/*k*-mer).

A super-*k*-mer comprises at most k−m+1*k*-mers by construction (Technically, there can be more than k−m+1 consecutive *k*-mers with the same minimizer. In this case, however, we can split the super-*k*-mer into chunks of at most k−m+1*k*-mers.). Let *j* be the starting position of a super-*k*-mer in *S*. The displacement function is therefore as follows.


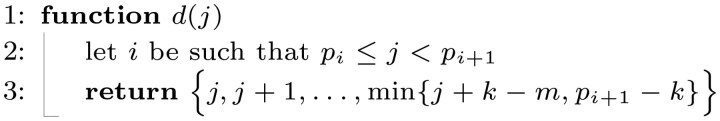


Since the index *i* is computed by d(j), the handle returned by SSHash at Line 8 of Algorithm 1 is h=q−(i−1)·(k−1), i.e. the *n* input *k*-mers are mapped to the minimal range *[*1.*n]*.

**Skew hashing.** Some minimizers ϕ may be very repetitive in *S*, hence making the set |loc(ϕ)| very large, and Lookup slow in the worst case. Fortunately, using a random order and a sufficiently long minimizer length *m*, the fraction of such minimizers is very small. This is referred to as the *skew* property of minimizer occurrences. We can therefore afford to handle these few minimizers with a more space-consuming data structure that, on the other hand, guarantees a better worst-case runtime. SSHash adopts the following solution.

Let *l* and *r* be two integers, such that 0≤l<r. Let $K_{i}=\{x\in {\text{spect}}_{k}(S)|2^{i}<|\text{loc}(\phi )|\leq \min\{2^{i+1},\max\},\phi =\Phi (x)\}$ for l≤i≤r, where $\max=\max_{\phi \in M}|\text{loc}(\phi )|$. By virtue of the skew property of minimizers, we have that $\sum |K_{i}|$ is a small fraction of the total *k*-mers. For example, we have that only ≈1.3% of the total *k*-mers belong to such sets, for the whole human genome with k=31, m=21, and l=6. An MPHF $f_{i}$ is built for each set $K_{i}$. For *k*-mer $x\in K_{i}$ with ϕ=Φ(x), we store the super-*k*-mer identifier among [1..|loc(ϕ)|] at position $f_{i}(x)$ in an array $V_{i}$. It follows that an integer in $V_{i}$ needs i+1 bits (or ${\;\text{log}\;}_{2}(\max)$ bits if i=r). For the rest of the paper, we refer to a pair $\left(f_{i},V_{i}\right)$ for l≤i≤r as a *partition* of the skew index. We have r−l+1 partitions (at most, when $\max\geq 2^{r}$).

At query time, if $|\text{loc}(\phi )|>2^{l}$ then the super-*k*-mer identifier is retrieved from $f_{i}$ and $V_{i}$ and the query *k*-mer searched *only in one* super-*k*-mer, instead of |loc(ϕ)| super-*k*-mers.

**Index/query modalities.** SSHash operates in two modalities, with different space/time trade-offs. In its *regular* modality, Φ=MINI, hence if LOOKUP(x)=⊥ then also $L\text{OOKUP}(\bar{x})$ is executed. In the worst case, both loc(MINI(x)) and $\text{loc}(M\text{INI}(\bar{x}))$ are inspected. In the *canonical* modality, Φ=CMini, so that a *k*-mer *x* and its reverse complement $\bar{x}$ are both mapped to the same set loc(CMini(x)). The Lookup algorithm is therefore executed only once (the boolean expression in the **if** at Line 8 of Algorithm 1 becomes: $x=y\;\mathrm{or}\;\bar{x}=y$) for faster query times compared to regular indexing at the price of some more space due to the higher density of canonical minimizers.

**Data structures and analysis.** The string *S* is a 2-bit integer vector, as each DNA base can be coded with 2 bits. The array *P*, indicating the start position of each substring $s_{i}$ in *S*, is coded with Elias-Fano and takes at most EF(|S|,N)+o(|S|) bits as per Theorem 1 (Points 1. and 2.). The locate table *50* is an array of ${\;\text{log}\;}_{2}(N)$-bit integers, where all locate sets are stored consecutively, one after the other, in the order indicated by the MPHF *f*. Let $Z=\sum_{\phi \in M}|\text{loc}(\phi )|$. The space of *L* is therefore $Z{\;\text{log}\;}_{2}(N)$ bits. The space for *f* is Θ(M) bits. With another array sizes[1..M+1] we keep track of where each loc(ϕ) begins and ends in *L*. That is, if t=f(ϕ), then loc(ϕ)=L[sizes[t]..sizes[t+1]), with |loc(ϕ)|=sizes[t+1]−sizes[t]. The sorted array *sizes* is also represented with Elias-Fano and takes at most EF(M,Z)+o(M) bits (Theorem 1, Point 1.). Letting $\alpha =\sum |K_{i}|$, we upper bound the cost of the skew index by $\alpha {\;\text{log}\;}_{2}(N)+\Theta (\alpha )$ bits, because a MPHF is built over each $K_{i}$ and ${\;\text{log}\;}_{2}(\max)<{\;\text{log}\;}_{2}(N)$. We thus have the following result.

Theorem 2. (Previous SSHash) The space usage of SSHash is at most $2N+(Z+\alpha ){\;\text{log}\;}_{2}(N)+\Theta (M)+\Theta (\alpha )+\text{EF}(|S|,N)+\text{EF}(M,Z)$ bits. The number of cache misses per LOOKUP(x), where z=|loc(MINI(x))|, is at most

1. $1+C_{\text{acc}}+C(z{\;\text{log}\;}_{2}(N))+z(C_{\text{succ}}+C(4k-2m))$ if $1\leq z\leq 2^{l}$,
2. $4+C_{\text{acc}}+C_{\text{succ}}+C(4k-2m)$ otherwise,

where $C_{\text{acc}}=3+C(O({\;\text{log}\;}^{4}M))$, $C_{\text{succ}}=2+C(O({\;\text{log}\;}^{4}|S|))+C_{\text{scan}}$, and $C_{\text{scan}}=C(\frac{N}{\left|S\right|})+C((\frac{N}{\left|S\right|}+1){\;\text{log}\;}_{2}(\frac{N}{\left|S\right|}))+C(\Delta_{P})$.

Figure S2 in the Supplementary Material shows that the space upper bound given in Theorem 2 is quite tight. (Most of the difference between the bound and the measured space comes from overestimating the cost of the skew index.) Table S1 in the Supplementary Material, instead, compares the number of theoretical cache misses in the theorem with the measured ones in practice (and shows that they are very similar).

## 5 Refining displacements

The first refinement we introduce in the updated SSHash index regards the elements of the set loc(ϕ), where ϕ is a minimizer. As explained in Section 4, in the previous version of SSHash, the elements of loc(ϕ) are the start positions of each super-*k*-mer whose minimizer is ϕ. While this works seamlessly for both the regular and canonical minimizer, it also involves a linear search of a super-*k*-mer upon Lookup. To avoid the search, we now let $\text{loc}(\phi ):=\{\text{pos}(x,S)+\text{pos}(\phi ,x)-1|x\in {\text{spect}}_{k}(S)\wedge \Phi (x)=\phi \}$.

**Regular displacements.** At query time, given j∈loc(ϕ), we compute the putative start position of *x* in *S* as q=j−pos(MINI(x),x)+1. That is, the displacement function now returns a single position, thus avoiding the need to actually search for the *k*-mer *x* in a super-*k*-mer.


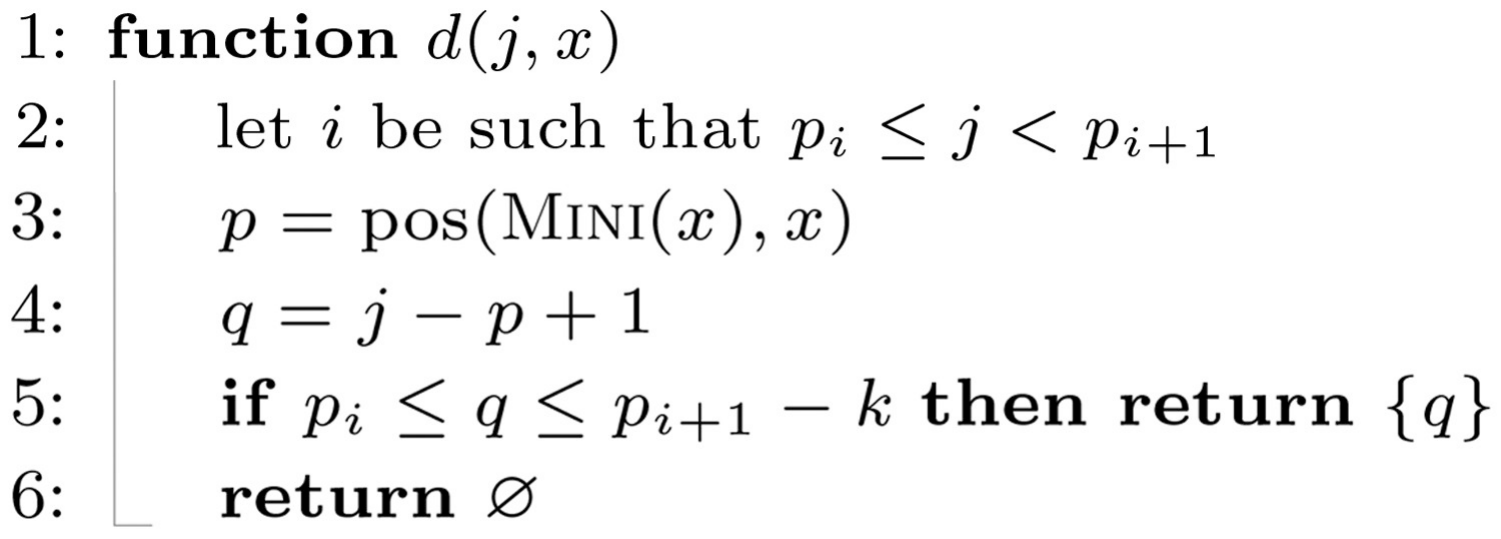


Refer to the string *S* shown in Fig. 1, panel (a), and consider the query *k*-mer x=AACTTGA. As explained in Section 4, if LOOKUP(x)=⊥ then $L\text{OOKUP}(\bar{x})$ is executed. We have MINI(x)=AAC and pos(MINI(x),x)=1. Note that no *k*-mer of *S* has minimizer AAC, hence f(AAC) indicates a locate set loc(ϕ) for another minimizer ϕ≠AAC, say ATC which begins in *S* at position j=27. The position q=27−1+1=27 is computed but y=S[27..27+k)≠x. Thus, $L\text{OOKUP}(\bar{x})$ is executed, where $\bar{x}=\mathrm{TCAAGTT}$. Now, $M\text{INI}(\bar{x})=\mathrm{AAG}$ and it begins at position 3 in $\bar{x}$. The candidate position q=3−3+1=1 is computed and $\bar{x}$ is compared against S[1..1+k) and produces a match.

**Canonical displacements.** The canonical indexing case, where Φ=CMini, is more involved. Recall from Section 2 that we defined the canonical minimizer of a *k*-mer *x* as $\text{CMini}(x):=\min\{M\text{INI}(x),M\text{INI}(\bar{x})\}.$ To let the new definition of loc(ϕ) work correctly, we also define


$$\text{pos}(\text{CMini}(x),x):=\{\begin{array}{cc} \text{pos}(M\text{INI}(x),x),\; & \text{if}\;M\text{INI}(x)\leq M\text{INI}(\bar{x})\\ \text{pos}(\bar{M\text{INI}(\bar{x})},x),\; & \text{otherwise} \end{array}$$

For example, consider x=TCAAGTT with $\text{CMini}(x)=M\text{INI}(\bar{x})=\mathrm{AAC}$. We have that pos(CMini(x),x)=5 because $\bar{\mathrm{AAC}}=\mathrm{GTT}$ begins at position 5 in *x*.

Now, given a query *k*-mer *x*, we do not know whether it appears in *S* as *x* or as $\bar{x}$ (or, if it appears at all). We therefore have to check more than one displacement per position j∈loc(CMini(x)) in the worst case:

1. If $M\text{INI}(x)<M\text{INI}(\bar{x})$:
  -
j−pos(MINI(x),x)+1 because *x* can appear in *S*;
  -
$j-\text{pos}(\bar{M\text{INI}(x)},\bar{x})+1$ because $\bar{x}$ can appear in *S* and MINI(x) occurs in $\bar{x}$ as $\bar{M\text{INI}(x)}$.
2. If $M\text{INI}(x)>M\text{INI}(\bar{x})$:
  -
$j-\text{pos}(M\text{INI}(\bar{x}),\bar{x})+1$ because $\bar{x}$ can appear in *S*;
  -
$j-\text{pos}(\bar{M\text{INI}(\bar{x})},x)+1$ because *x* can appear in *S* and $M\text{INI}(\bar{x})$ occurs in *x* as $\bar{M\text{INI}(\bar{x})}$.
3. If $M\text{INI}(x)=M\text{INI}(\bar{x})$: all the four displacements are checked.

Observation 1. Let ϕ be a substring of length m of a k-mer x. Then $\text{pos}(\bar{\phi },\bar{x})=k-m-\text{pos}(\phi ,x)+2$.

From the previous case analysis and Observation 1, we derive the following displacement function.


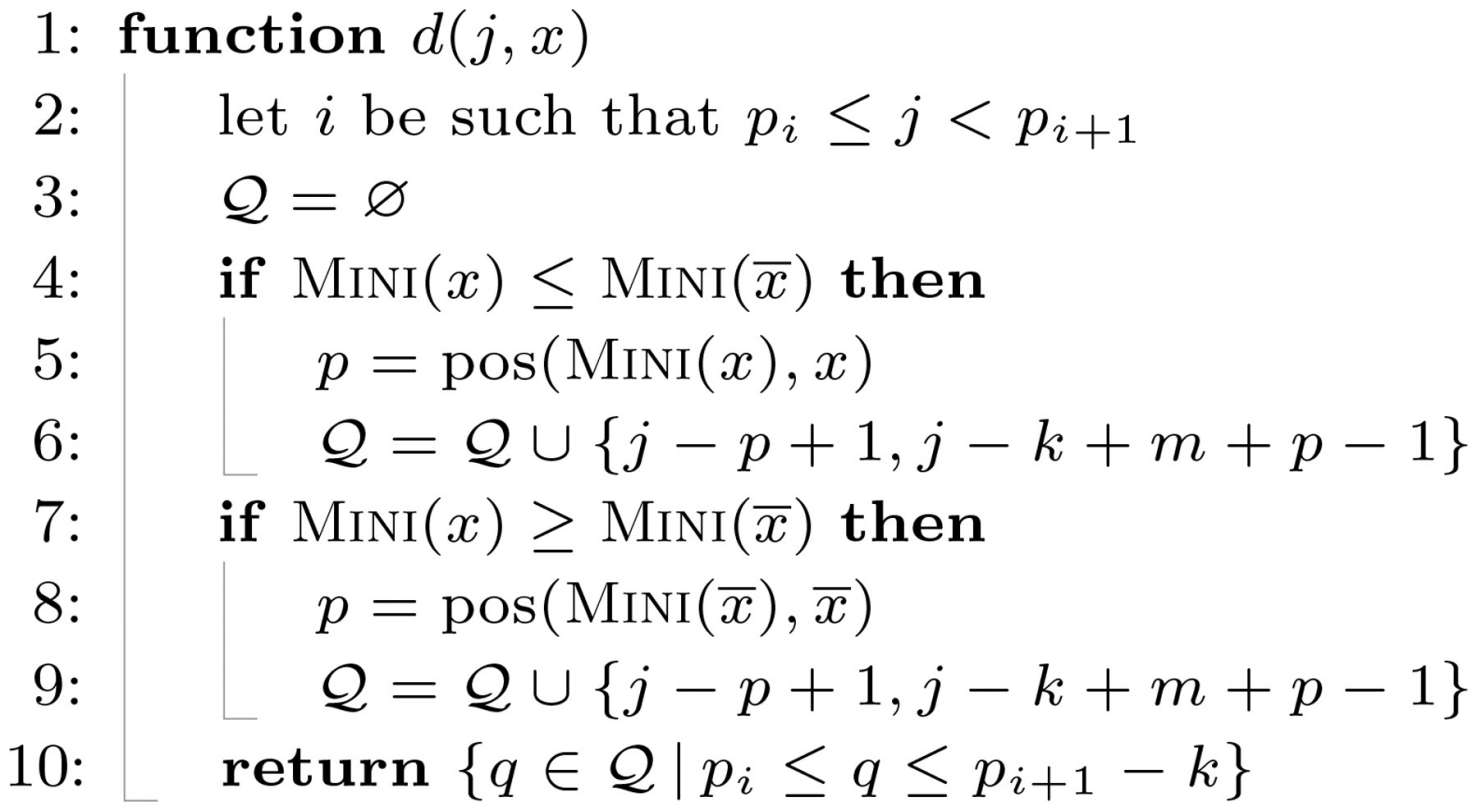


Note that for Cases 1 and 2, just two displacements are computed. For Case 3, four displacements are computed. Some of them might not be valid and not returned at all (Line 10).

Consider again the string *S* in Fig. 1, panel (b). Suppose we query for x=TTGGCCT. We have $M\text{INI}(\bar{x})<M\text{INI}(x)$ and $M\text{INI}(\bar{x})=\mathrm{AGG}$, which begins at position 1 in $\bar{x}=\mathrm{AGGCCAA}$. The minimizer AGG appears in *S* as $\bar{\mathrm{AGG}}=\mathrm{CCT}$ at position j=10. The two positions to check, returned by d(x,j), are therefore {10,6} and *x* is found at position 6 in *S*.

Lastly, the following observation will be useful for Theorem 3 in the analysis from Section 6.

Observation 2. The k-mers starting at positions d(j,x) occur in the same substring of S and this substring is at most 2k−m characters long. Hence, accessing S at positions in d(j,x) in ascending order costs C(4k−2m) cache-misses.

## 6 Cache-efficient sparse hashing

In this section, we present a second refinement: a new data structure for sparse hashing that reduces the number of cache misses involved during Lookup.

We distinguish three *types* of minimizers based on their number of occurrences in *S*: we call a minimizer *singleton* if it appears once, *light* if it appears more than once but at most $2^{l}$ times for a small value of l≥1, and *heavy* otherwise (it appears more than $2^{l}$ times). To reduce cache misses during Lookup queries, the idea is to reorganize the storage of the locate sets loc(ϕ) into three arrays according to the type of each minimizer. Let *T[*1.*M]* be an array of $\left({\;\text{log}\;}_{2}(N)+1\right)$-bit integers, referred to as *tags* in the following, indexed by *f*. We use this array of tags to indicate the type of the minimizer and therefore retrieve its locate set from a dedicated array. This design allows Lookup to follow type-specific execution paths, thereby reducing cache misses. Retrieving the tag itself involves computing *f* and accessing T[f(ϕ)]: these two operations cost two cache misses.

- *Singleton minimizers.* If the minimizer appears once, the least significant bit of the tag is 0 and the remaining ${\;\text{log}\;}_{2}(N)$ bits encode the single position where the minimizer appears in *S*. At query time, whenever we read status bit 0, we know the minimizer is singleton and the only position in the locate set is readily available in the tag without any further memory access.
- *Light minimizers.* All locate sets of size $2\leq z\leq 2^{l}$ are stored contiguously in an array *L*, grouped by increasing size. We maintain a small auxiliary array $G[1..2^{l}]$, where *G[z]* stores the starting position in *L* of the group containing sets of size *z*. For a light minimizer ϕ such that z=|loc(ϕ)|, the tag consists of (from least to most significant bits): a 2-bit status field equal to 01; *l* bits encoding the value z−2; ${\;\text{log}\;}_{2}(N)-l-1$ bits encoding the position, *i*, of loc(ϕ) in the group of all sets of size *z* (Technically speaking, we should choose *l* so that the number of minimizers with two occurrences is less than $2^{{\;\text{log}\;}_{2}(N)-l-1}$. Due to the skew distribution of such occurrences for sufficiently long *m*, this is always the case for l=6 across all of our experiments.). At query time, we decode *z* and *i* from the tag and access loc(ϕ)=L[G[z]+iz..G[z]+(i+1)z). Retrieving *G[z]* costs 1 cache miss. The number of cache misses involved during a scan of loc(ϕ) are $C(|\text{loc}(\phi )|{\;\text{log}\;}_{2}(N))\leq C(2^{l}{\;\text{log}\;}_{2}(N))$.
- *Heavy minimizers.* Minimizers occurring more than $2^{l}$ times are assigned status 11 and their locate sets are stored in another array *H*. These minimizers are handled with a skew index. Recall from Section 4 that a skew index comprises a collection of (at most) r−l+1 pairs $\left(f_{i},V_{i}\right)$, where $f_{i}$ is a MPHF and $V_{i}$ is an vector of (i+1)-bit integers. Here, we choose *r* so that r−l+1≤8 and a skew index partition identifier *i* can be coded in 3 bits. For a heavy minimizer ϕ, the corresponding tag stores: a 2-bit status equal to 11; 3 bits encoding the skew partition identifier *i*; the remaining ${\;\text{log}\;}_{2}(N)-4$ bits encoding the absolute offset *o* of loc(ϕ) in *H*. Assume ϕ=Φ(x) is a heavy minimizer. After recovering *i* and *o* from its tag, the desired position j∈loc(ϕ) is obtained as $j=H[o+V_{i}[f_{i}(x)]]$, involving 3 cache misses.

**Figure 2:**
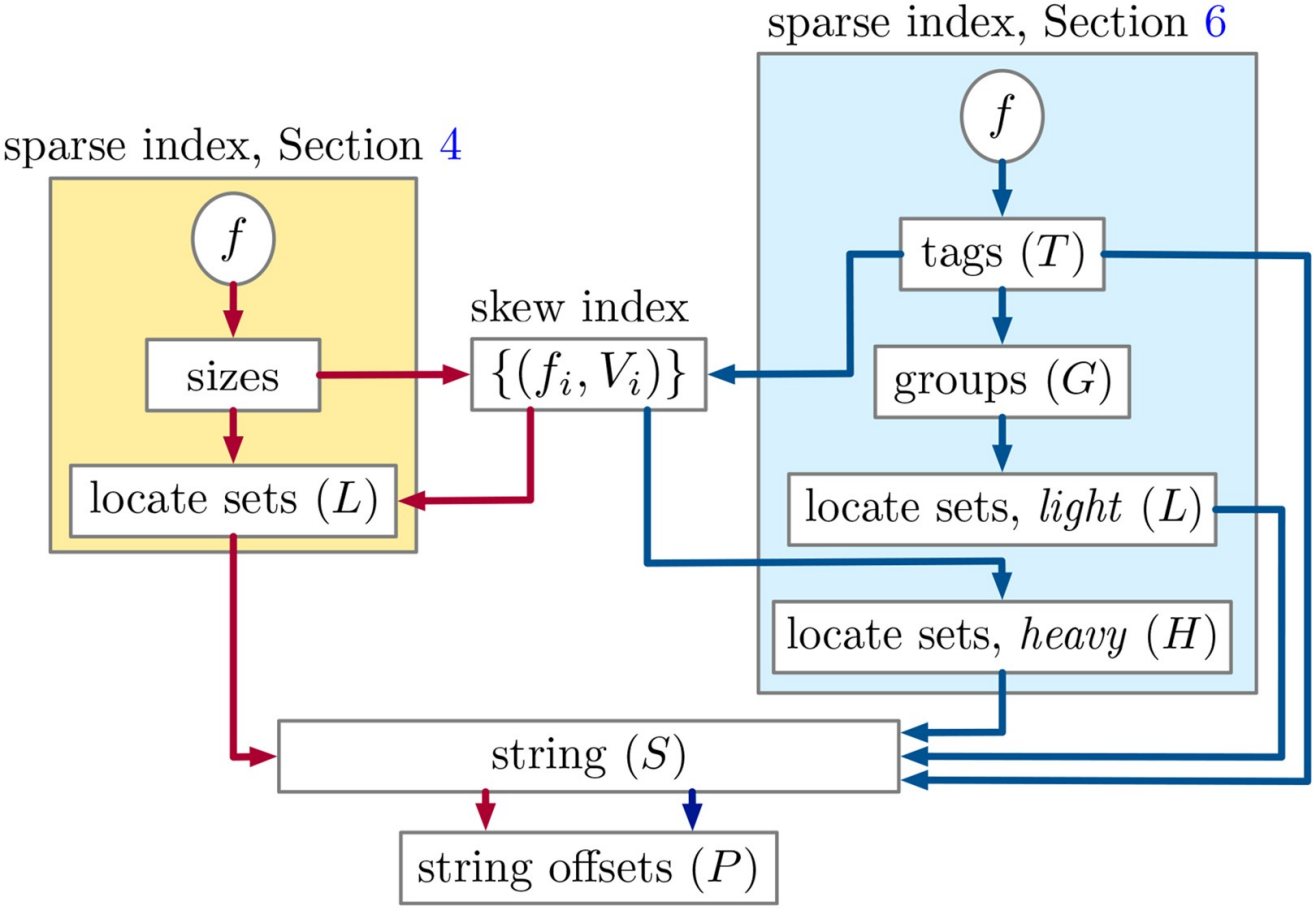
A graphical comparison between the components of the two versions of SSHash described in Section 4 and Section 6 (symbolic names used in the text are reported in parentheses). The skew index, *S*, and *P*, are common to both versions. In this (multi) graph, a path from *f* to *P* corresponds to the flow of execution upon Lookup (with the constraint that edges along the path do not change color). Note, in fact, that there are two possible red paths for the previous index (corresponding to the cases of Theorem 2), and three blue paths for the current one (cases of Theorem 3).

Figure 2 illustrates how this layout differs from the previous one described in Section 4.

**Analysis.** Let β∈[0,1] be the fraction of minimizers that are not singleton (the number of singleton minimizers is (1−β)M). The array of tags takes $M({\;\text{log}\;}_{2}(N)+1)$ bits, whereas the arrays *L* and *H* take $\left(Z-(1-\beta )M){\;\text{log}\;}_{2}(N\right)$ bits (the array *G* is a global redundancy, which is negligible for small *l*, even if stored uncompressed as we do). The other costs are those for *S* and *f*, which are the same as those in Theorem 2, and *P* that we now represent with the data structure from Theorem 1, Point 3. Importantly, we eliminate the *sizes* array altogether. While this does not affect space too much, it is rather relevant to reduce the number of cache misses (see the next theorem). Considering the refined displacements from Section 5 and the new layout in this section, we obtain the following result.

Theorem 3. (Current SSHash) The space usage of SSHash is at most $2N+(Z+\alpha ){\;\text{log}\;}_{2}(N)+M(1+\beta {\;\text{log}\;}_{2}(N))+\Theta (\alpha )+\Theta (M)+O(|S|\;\text{log}\;|S|)+\text{EF}(|S|,N)+\text{EF}(M,Z)$ bits. The number of cache misses per LOOKUP(x), where z=|loc(MINI(x))|, is at most

1. $2+C_{\text{succ}}+C(2k)$ if z=1,
2. $3+C(z{\;\text{log}\;}_{2}(N))+z(C_{\text{succ}}+C(2k))$ if $2\leq z\leq 2^{l}$,
3. $5+C_{\text{succ}}+C(2k)$ otherwise,

where $C_{\text{succ}}=1+C_{\text{scan}}$ and $C_{\text{scan}}$ is the same as that in Theorem 2. When Φ=CMini, the cost C(2k) in Case 2. becomes C(4k−2m) due to Observation 2.

Assuming the same constants hidden in the Θ terms of Theorems 2 and 3, e.g. by using the same MPHF data structure, the space increase of Theorem 3 over Theorem 2 is less than $M(\beta {\;\text{log}\;}_{2}(N)-{\;\text{log}\;}_{2}(Z/M)-1)+O(|S|\;\text{log}\;|S|)$ bits. This space is small when β decreases as *m* increases. For example, β is on average 0.053 for k=31 and m≥19, Φ=MINI, on the datasets used in our analysis in Section 8 (see also Figs. S1 and S2 in the Supplementary Material).

For the cache miss analysis in practice, refer again to Section 2 and Table S1 in the Supplementary Material.

## 7. Streaming query algorithm

Finally, we describe the simplified streaming query algorithm we adopt in the improved SSHash index. Let *s* be a string with |s|≥k. We consider the query LOOKUP(s), from Problem 1, that answers LOOKUP(x) for all *k*-mers x∈s.

Given that two consecutive *k*-mers of *s*, say *w* and *x*, share a (k−1)-long suffix-prefix overlap, answering LOOKUP(x)*after* LOOKUP(w) should be performed faster than issuing the queries for two *k*-mers of *s* picked in any order. That is, a good implementation of LOOKUP(s) should *stream* through the *k*-mers of *s* to exploit the overlap information and, hence, accelerate the query.

Algorithm 2 shows the solution we use in SSHash. The general idea is to maintain the *state* of the last match *w* and use this information to perform faster pattern matching. For example, if the last match was found at position *q* in *S*, the next query, say for *k*-mer *x*, will compare *x* (or $\bar{x}$) to S[q+1..q+1+k). The state of the last match *w*, is made up of several variables: its handle *h*, the identifier *i* of the comprising string, its orientation o∈{−1,+1} in *S* (under the convention that +1 indicates the forward orientation, and −1 indicates the backward orientation), whether its minimizer was *found* in *S*, its position *q* in *S*, and its minimizers MINI(w) and $M\text{INI}(\bar{w})$. (Note that the tuple (h,i,o,found), computed at Line 30, can be returned by the Lookup algorithm with a straightforward modification of Algorithm 1.)

The other two variables that are part of the state are the boolean flag *start* and the integer *budget*. The *start* variable is used to let the function Minimizers (Line 14) compute correctly the minimizers of *x knowing* those of the last match *w*: that is, if start=true then *w* is not defined and the pair $\left(M\text{INI}(x),M\text{INI}(\bar{x})\right)$ is computed from scratch; otherwise, the pair is computed in amortized O(1) time (using, e.g. the folklore *re-scan* method; see, the discussion in Koerkamp and Martayan (2025) and Zheng *et al.* (2025). The integer *budget* defines the maximum allowable number of extensions starting from position *q* in *S*. As we extend, we decrease the budget (Line 18–21). When the budget is exhausted, we update the state of the algorithm with the function Seed.

This logic is, *essentially*, the same as that described in the prior SSHash work (Section 4.3 of Pibiri (2022) but *simpler*, thanks to the *budget* “trick.” The previous algorithm attempts to extend only if the minimizers of the last match *w* are the same as those of the current query *x*, and calls Seed whenever they change. While this already grants a fair deal of extension (because consecutive *k*-mers are likely to have the same minimizer), in the logic presented here, we attempt an extension whenever budget>0—even when minimizers change through the stream. We show in the Supplementary Material (Fig. S4) that the extension rate of this refined logic is consistently higher than the previous one, granting faster query times. The two refinements presented in Sections 5 and 6 have an important impact on the runtime of this streaming query algorithm as well.

**Algorithm 2** The Lookup(s) algorithm for a query string *s*. In the pseudo code, we assume an “iterator-like” interface for the string *S* such that: S.ATq instantiates the iterator at position *q* of *S*, and S.NEXT moves the iterator from the current position to the next and returns the *k*-mer at such position (taking into account the orientation *o* of the last match).

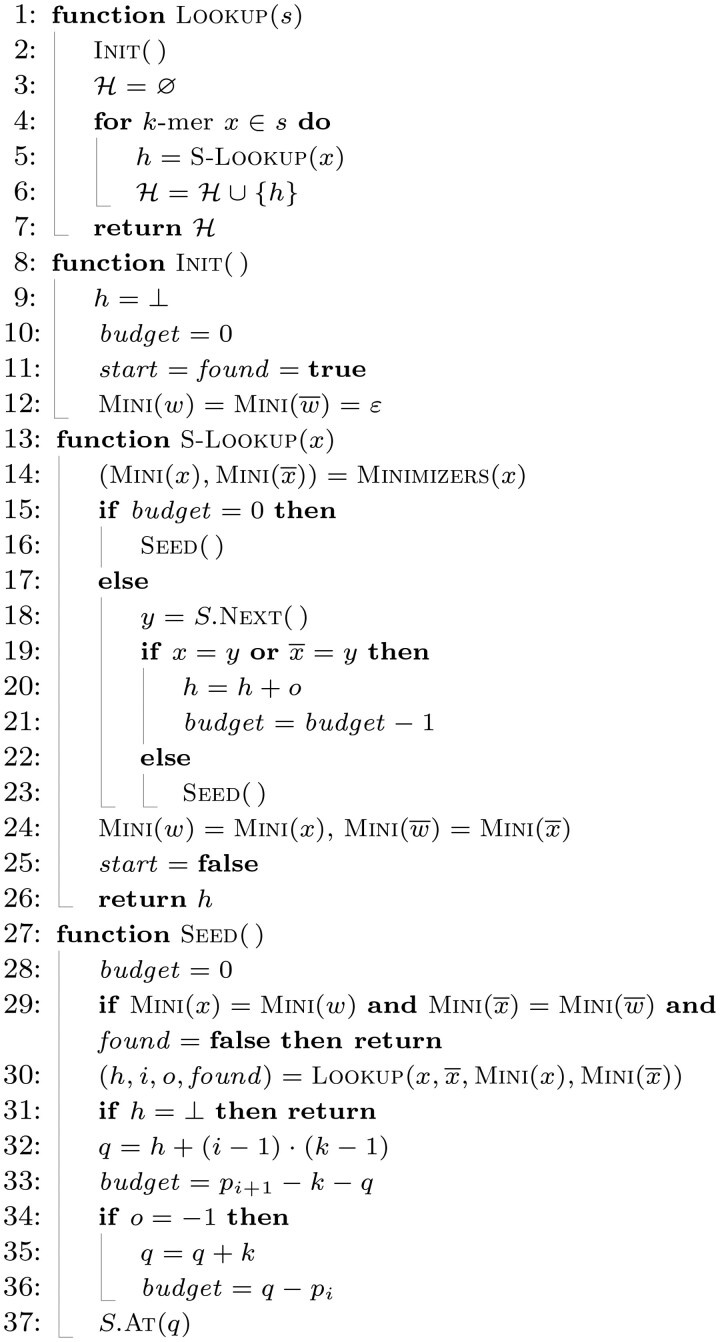

## 8 Experimental analysis

In this section, we compare the new SSHash design against the two state-of-the-art *k*-mer dictionaries, SBWT (Alanko *et al.* 2023a) and FMSI (Sladký *et al.* 2026). (The Supplementary Material also reports on the comparison against the previous published version of SSHash (Pibiri 2022), using the same methodology and datasets described in this section.)

In particular, we report on the results of the experiments that were collected during November 2025 with the help of the authors of both the SBWT and FMSI. We maintain the benchmark at https://github.com/jermp/kmer_sets_benchmark, to encourage reproducibility of results. The scripts available at the repository list the precise options we used for the tools; we just report some details here.

The SBWT was always built by indexing both *k*-mer strands as to accelerate query processing, using the so-called “plain-matrix” variant. This is the recommended usage by the authors (and the one used in their tool Themisto (Alanko *et al.* 2023b)—an index for colored De Bruijn graphs based on the SBWT). Both SBWT and FMSI indexes make use of the *longest common prefix* (LCP) array to speed up queries. The space for this additional array is also included in the reported space usage.

**Hardware and compiler.** All experiments were executed on a machine equipped with a AMD Ryzen Threadripper PRO 7985WX CPU, 250 GB of RAM, and a Seagate IronWolf 12 TB NAS HDD, running Ubuntu 24.04.3 LTS. All software is written in C++ and was compiled with gcc 13.3.0 using the highest optimization setting (compiler options: -O3 -march=native).

**Datasets.** For the experiments reported here we used two types of datasets: whole genomes and pangenomes. For the former type and for consistency with prior published work, we used the whole genomes of *Gadus morhua*, *Falco tinnunculus*, and *Homo sapiens* that are named Cod, Kestrel, and Human, respectively in the following (containing approx. 0.5, 1.5, and 2.5 billion distinct *k*-mers). For the latter type, we used: NCBI-v—a collection of 18,836 virus assemblies downloaded from RefSeq in October 2025 (approx. 0.4 billion *k*-mers); SE—a pangenome containing all the 534,751 *Salmonella enterica* genomes from Release 0.2 of the “All The Bacteria” collection (Hunt *et al.* 2025) (approx. 0.9 and 1.5 billion *k*-mers for k=31 and k=63, respectively); HPRC—a human pangenome available at https://zenodo.org/records/14854401 (approx. 3.7 and 5.9 billion *k*-mers for k=31 and k=63, respectively). From these input collections, we computed spectrum-preserving string sets in the form of eulertigs (Schmidt and Alanko 2023) using the GGCAT algorithm (Cracco and Tomescu 2023). These eulertigs, along with detailed instructions about how we computed them, are available at https://zenodo.org/records/17582116, so that it is easy to reproduce our results.

Table S2 in the Supplementary Material reports the exact number of unique *k*-mers for the two values of *k* we use in this analysis, and the minimizer lengths used by SSHash.

**Figure 3:**
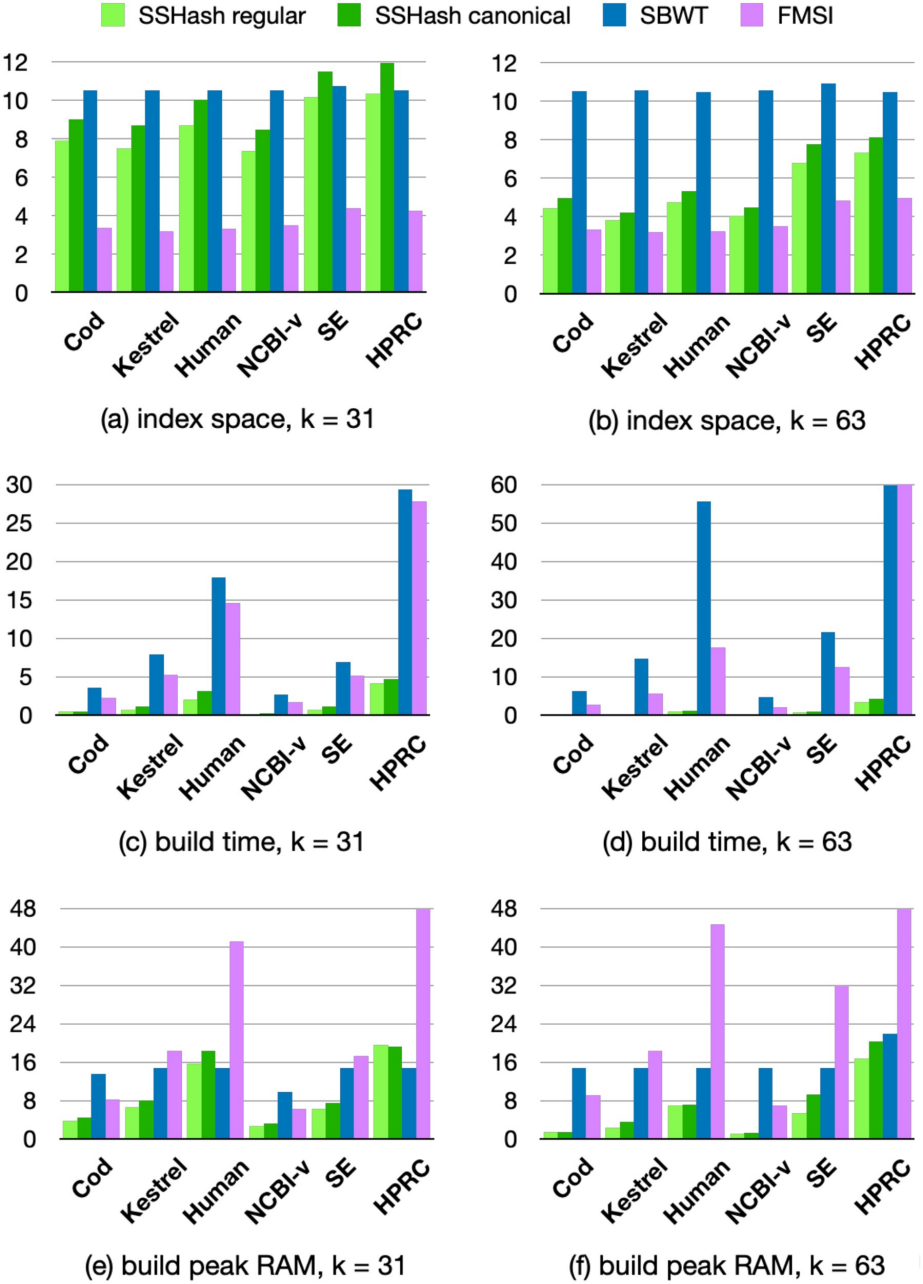
Comparison of index space in avg. bits/*k*-mer (plots (a)-(b)), build time in minutes (plots (c)-(d)), and build peak RAM usage (resident set size, or RSS) in GiB (plots (e)-(f)). In plot (d), we cut the bars for SBWT at 60 minutes for ease of visualization because it took three hours to build on HPRC. (FMSI was built in 62 minutes.) Similarly, we cut the bars for FMSI in plots (e)-(f) at 48 GiB because it required 70 and 125 GiB of RAM on HPRC for k=31 and k=63, respectively.

**Index space.** Plots (a) and (b) of Fig. 3 report index space in avg. bits/*k*-mer. FMSI is consistently the smallest index, taking between 3.3 and 5.0 bits/*k*-mer, whereas the SBWT has a steady usage of 10.5 bits/*k*-mer. SSHash is competitive with the space usage of SBWT, and its space lowers substantially for larger *k* as minimizers become sparser. For this reason, in some cases like Kestrel and NCBI-v for k=63, it is less than 1 bit per *k*-mer away from the effectiveness of FMSI.

**Construction efficiency.** All construction algorithms read the input files from the (mechanical) disk of the testing machine. SSHash used a maximum of 16 GiB of RAM during construction and 64 threads. The same configuration was used for SBWT, whereas FMSI’s construction does not accept such parameters.

We explicitly clarify that the build times reported here strictly measure the *construction of the indexes*. They do not encompass the time required for upstream preprocessing steps, such as the construction of masked super-strings for FMSI or the generation of eulertigs for SSHash. It should be therefore clear that our plots do not represent the total end-to-end operational cost starting from raw datasets.

Plots (c) and (d) of Fig. 3 report the time to build the indexes. Even on the largest collections, SSHash completed within 5 minutes, whereas the other tools took up to 1 hour. (For FMSI, we do not include the time it takes to pre-process the input to obtain the so-called *masked super-string* that it indexes.) SBWT is slower than FMSI especially for larger *k* because it enumerates and sorts *k*-mers co-lexicographically on disk (for both strands).

Lastly, plots (e) and (f) display the peak RAM usage (in GiB) during index construction. Both SSHash and SBWT generally remain within the allocated 16 GiB memory budget, except when processing the HPRC dataset. For SSHash, this exception occurs because the final HPRC index occupies an additional 5.6 GiB of memory on top of the 16 GiB construction buffer. In contrast, the construction phase of FMSI does not currently support bounding RAM usage.

**Query efficiency.** We say LOOKUP(x) is *positive* if *k*-mer *x* is found in the dictionary (indicated with ${L\text{OOKUP}}^{+}$ in the plots) and *negative* otherwise (indicated with ${L\text{OOKUP}}^{-}$). To benchmark positive Lookup, $10^{6}$*k*-mers were sampled uniformly at random from the collections and used as input. Importantly, half of them were reverse complemented to test the Lookup algorithm in the most general case. To benchmark negative Lookup instead, $10^{6}$ synthetic *k*-mers were created, having characters selected uniformly at random from the alphabet {A,C,G,T}. For Access, we generated $10^{6}$ random handles and retrieved the *k*-mers corresponding to those handles. The reported times are the averages among five runs of the same experiment.

To benchmark streaming Lookup queries, we take as input all the reads from FASTQ files, also available in the data repository at https://zenodo.org/records/17582116. These files, that contain millions of reads and are compressed with gzip, are decompressed on the fly while performing the queries. The reported time therefore also includes the incremental decompression overhead which, however, is marginal and paid by all the tested tools. The reads were chosen for each collection as to simulate a “high-hit” workload, i.e. where most *k*-mers are present in the index (say, more than 75% for k=31).

All query algorithms were run using a single processing thread. All indexes were loaded from disk to RAM after construction to perform the queries.

**Figure 4:**
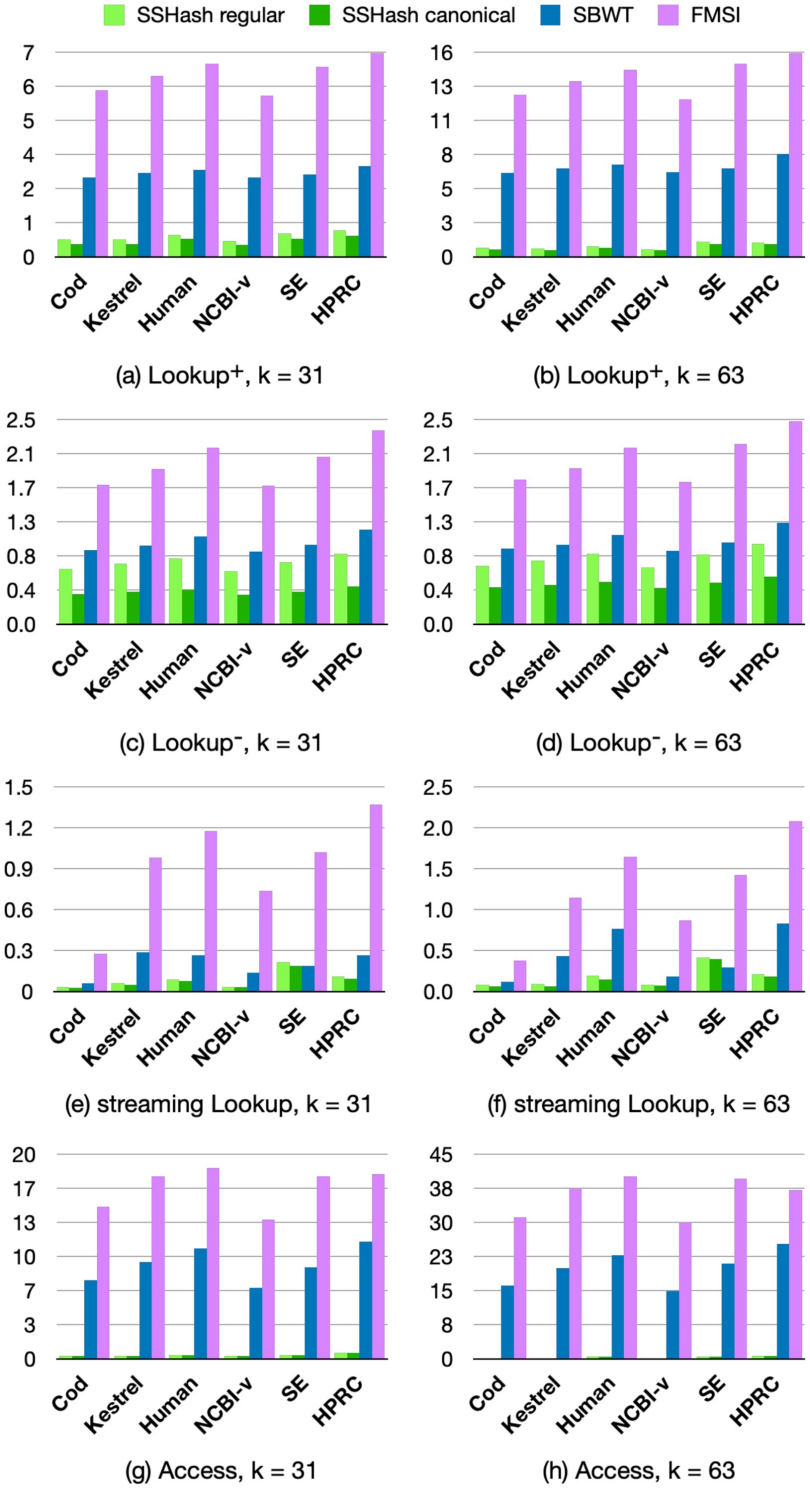
Comparison of query times in avg. μs/*k*-mer.

Figure 4 illustrates the comparison between query times. SSHash is generally the fastest index for all queries, by a wide margin. FMSI is the smallest index on disk as said before, but also the slowest to query. For example, the SBWT is 2× faster than FMSI for random Lookup queries and much faster at streaming queries. Remarkably, SBWT is even faster than SSHash on the SE dataset for streaming queries, k=63. The Access query is problematic for indexes based on the BWT, as it requires tens of microseconds whereas SSHash takes a fraction of a microsecond.

In summary, SSHash regular is on average 4.2, 1.3, and 2.9 faster than the next fastest index (SBWT) for positive, negative, and streaming queries, respectively, for k=31; and 8.7, 1.2, 2.9, respectively for k=63. (These factors are higher for SSHash canonical: 5.5, 2.5, 3.5 for k=31; 10, 2, 3.6 for k=63).

## 9 Conclusions and future work

We presented an improved sparse and skew hashing design for the *k*-mer dictionary problem, featuring a cache-efficient layout, simpler queries, and faster streaming lookups. These speed improvements are expected to accelerate downstream processing, including indexing weighted/colored De Bruijn graphs and spectrum-preserving tilings.

Compared to other indexes based on the celebrated Burrows-Wheeler transform, we found SSHash to be faster to query and build, but to generally consume more space. Batch processing mitigates memory latency in compressed indexes. For instance, batched *k*-mer lookups significantly improve SBWT throughput (Alanko *et al.* 2025). While SSHash would also benefit, BWT-based indexes likely gain a greater relative advantage.

Future work will study the applicability of different string sampling schemes for SSHash. For example, preliminary experiments show that *mod-minimizers* (Koerkamp *et al.* 2025) have the potential to reduce space consumption without hurting lookup time. Another direction could replace the locally-consistent sampling of minimizers with a *sequence-specific* one. The latter has the promise of achieving optimal density but its impact on query time, on the other hand, has yet to be analyzed.

## Supplementary Material

btag264_Supplementary_Data

## Data Availability

The data underlying this article are available at https://zenodo.org/records/17582116.

## References

[btag264-B1] Alanko JN , Biagi E, Mackenzie J et al Batched-mer lookup on the spectral burrows-wheeler transform. In: *ALENEX*, pp. 95–106, 2025.

[btag264-B2] Alanko JN , Puglisi SJ, Vuohtoniemi J. Small searchable κ-spectra via subset rank queries on the spectral Burrows-Wheeler transform. In: *ACDA*, pp. 225–36, 2023a.

[btag264-B3] Alanko JN , Vuohtoniemi J, Mäklin T et al Themisto: a scalable colored *k*-mer index for sensitive pseudoalignment against hundreds of thousands of bacterial genomes. Bioinformatics2023b;39:i260–9. 37387143 10.1093/bioinformatics/btad233 PMC10311346

[btag264-B4] Almodaresi F , Sarkar H, Srivastava A et al A space and time-efficient index for the compacted colored de Bruijn graph. Bioinformatics 2018;34:i169–77. 29949982 10.1093/bioinformatics/bty292 PMC6022659

[btag264-B5] Bingmann T , Bradley P, Gauger F et al COBS: a compact bit-sliced signature index. In: *SPIRE*, pp. 285–303, 2019.

[btag264-B6] Břinda K , Baym M, Kucherov G. Simplitigs as an efficient and scalable representation of de Bruijn graphs. Genome Biol 2021;22:96–24. 33823902 10.1186/s13059-021-02297-z PMC8025321

[btag264-B7] Burrows M , Wheeler D. A block-sorting lossless data compression algorithm. In: *Digital SRC Research Report*, 1994.

[btag264-B8] Campanelli A , Pibiri GE, Fan J et al Where the patterns are: repetition-aware compression for colored de Bruijn graphs. J Comput Biol 2024;31:1022–44. 39381838 10.1089/cmb.2024.0714 PMC11631793

[btag264-B9] Cracco A , Tomescu AI. Extremely fast construction and querying of compacted and colored de Bruijn graphs with GGCAT. Genome Res 2023;33:1198–207. 37253540 10.1101/gr.277615.122 PMC10538363

[btag264-B10] Fan J , Khan J, Pibiri GE et al Spectrum preserving tilings enable sparse and modular reference indexing. In: *RECOMB*, pp. 21–40, 2023.

[btag264-B11] Fan J , Khan J, Singh NP et al Fulgor: a fast and compact *k*-mer index for large-scale matching and color queries. Algorithms Mol Biol 2024;19:3. 38254124 10.1186/s13015-024-00251-9 PMC10810250

[btag264-B12] Hunt M , Lima L, Anderson D et al AllTheBacteria – all bacterial genomes assembled, available, and searchable, bioRxiv, 2025.

[btag264-B13] Koerkamp RG , Liu D, Pibiri GE. The open-closed mod-minimizer algorithm. Algorithms Mol Biol 2025;20:4. 40098006 10.1186/s13015-025-00270-0 PMC11912762

[btag264-B14] Koerkamp RG , Martayan I. SimdMinimizers: computing random minimizers, fast. In: *SEA*, pp. 20:1–20:19, 2025.

[btag264-B15] Lehmann H-P , Mueller T, Pagh R et al Modern minimal perfect hashing: a survey. ACM Comput Surv 2026;58:1–36.

[btag264-B16] Marchet C , Kerbiriou M, Limasset A. BLight: efficient exact associative structure for *k*-mers. Bioinformatics 2021;37:2858–65. 33821954 10.1093/bioinformatics/btab217

[btag264-B17] Mehlhorn K. On the program size of perfect and universal hash functions. In: *FOCS*, pp. 170–5, 1982.

[btag264-B18] Pibiri GE. Sparse and skew hashing of *k*-mers. Bioinformatics 2022;38:i185–94. 35758794 10.1093/bioinformatics/btac245 PMC9235479

[btag264-B19] Pibiri GE , Trani R. PTHash: Revisiting FCH minimal perfect hashing. In: *SIGIR*, pp. 1339–48, 2021.

[btag264-B20] Rahman A , Medvedev P. Representation of *k*-mer sets using spectrum-preserving string sets. In: *RECOMB*, pp. 152–68, 2020. 10.1089/cmb.2020.0431 PMC8066325 33290137

[btag264-B21] Schmidt S , Alanko JN. Eulertigs: minimum plain text representation of *k*-mer sets without repetitions in linear time. Algorithms Mol Biol 2023;18:5. 37403080 10.1186/s13015-023-00227-1 PMC10318842

[btag264-B22] Schmidt S , Khan S, Alanko JN et al Matchtigs: minimum plain text representation of k-mer sets. Genome Biol 2023;24:136. 37296461 10.1186/s13059-023-02968-z PMC10251615

[btag264-B23] Sladký O , Veselý P, Břinda K. From superstring to indexing: a space-efficient index for unconstrained *k*-mer sets using the Masked Burrows-Wheeler Transform (MBWT). Bioinform Adv 2026;6:vbaf290. 41542367 10.1093/bioadv/vbaf290 PMC12800775

[btag264-B24] Vitter JS. External memory algorithms and data structures: dealing with massive data. ACM Comput Surv 2001;33:209–71.

[btag264-B25] Zheng A , Lee I, Shivakumar VS et al Fast and flexible minimizer digestion with digest. Bioinformatics 2025;41:btaf368. 40581603 10.1093/bioinformatics/btaf368 PMC12233085

[btag264-B26] Zheng H , Kingsford C, Marçais G. Improved design and analysis of practical minimizers. Bioinformatics 2020;36:i119–27. 32657376 10.1093/bioinformatics/btaa472 PMC8248892

