# Supplementary material for: Optimizing sparse and skew hashing: faster k-mer dictionaries
Source: Bioinformatics. 2026 Jul 7;42(Suppl 1):btag264. doi: 10.1093/bioinformatics/btag264 (PMC13340181; doi:10.1093/bioinformatics/btag264)
Supplement: btag264_Supplementary_Data [file btag264_supplementary_data.pdf]

SUPPLEMENTARY MATERIAL

Optimizing sparse and skew hashing: faster k-mer dictionaries

Giulio Ermanno Pibiri[1] and Rob Patro[2]

[1]DAIS, Ca' Foscari University of Venice, Italy and [2]Dept. of Computer Science, University of Maryland, College Park, MD 20440, USA

Abstract

Supplementary Material for the paper "Optimizing sparse and skew hashing: faster k-mer dictionaries".

Contact: giulioermanno.pibiri@unive.it, rob@cs.umd.edu.

1. Elias-Fano sequences

Consider a sorted sequence A of n integers

$$0 \leq A[1] < A[2] < \cdots < A[n] < U$$

for some universe size U. The query $\textsc{Successor}(x)$ returns the smallest integer $y \in A$ that is $y \geq x$ (assuming, w.l.o.g., $x \leq A[n]$, so that the result is well-defined). We prove the following result based on Elias-Fano codes [Elias, 1974, Fano, 1971].

Theorem 1 (Elias-Fano) There exists a representation of A that takes at most $\text{EF}(n, U) = n(\ell + 3)$ bits and:

1. For $o(n)$ extra bits, access to the i-th element, can be supported with, at most, $1 + C_{\text{sel}}$ cache misses.
2. For $o(n)$ extra bits, $\textsc{Successor}$ can be supported with, at most, $C_{\text{sel}} + C_{\text{scan}}$ cache misses.
3. For $O(n \log n)$ extra bits, $\textsc{Successor}$ can be supported with, at most, $1 + C_{\text{scan}}$ cache misses.

where

- $C_{\text{sel}} = 2 + C(O(\log^4 n))$,
- $C_{\text{scan}} = C(U/n) + C(\ell \cdot (U/n + 1)) + C(\Delta_A)$,
- $\Delta_A := \max_{1 \leq i < n}\{\lfloor \frac{A[i+1]}{2^\ell} \rfloor - \lfloor \frac{A[i]}{2^\ell} \rfloor\}$,
- and $\ell = \lfloor \log_2(U/n) \rfloor$.

Representation. The binary $\lceil \log_2 U \rceil$-bit representation of each integer of A is split into two parts: its ℓ least significant bits and the remaining $h = \lceil \log_2 U \rceil - \ell$ most significant bits. We call these parts the *low* and *high* parts respectively. All the n low parts are written explicitly in a vector of ℓ-bit integers, whereas the high parts are coded using a bitvector A_{high} of length $n + \lfloor U/2^\ell \rfloor + 1$ bits, which is at most $3n$ bits because $n \leq \lfloor U/2^\ell \rfloor < 2n$. The main space bound, $\text{EF}(n, U) = n\lfloor \log_2(U/n) \rfloor + 3n$, follows.

The elements of A can be viewed as logically clustered into $\lfloor U/2^\ell \rfloor + 1$ clusters, $A_0, \ldots, A_{\lfloor U/2^\ell \rfloor}$, such that A_j contains the consecutive elements of A that have their high bits equal to j. The bitvector A_{high} is then

$$1^{|A_0|}0\ 1^{|A_1|}0\ 1^{|A_2|}0 \ldots 1^{|A_{\lfloor U/2^\ell \rfloor}|}0.$$

That is, it writes the cardinalities of the clusters in unary code. (Note that $|A_j|$ could be 0, i.e., no element in A has high bits equal to j. In this case, the unary code is a single 0 bit. Runs of zeros might be present in A_{high}. The length of the longest such run *between two consecutive ones* is Δ_A.) It follows that A_{high} has exactly n bits set.

Random access. To decode the i-th value, say $A[i] = x$, from the representation, the two parts must be re-linked together. Let x_ℓ and x_h be the low and high parts of x respectively, so that $x = x_h \cdot 2^\ell + x_\ell$. The low bits x_ℓ are read directly from the corresponding vector, spending one cache miss. The high bits x_h are computed by searching A_{high}, which can be done efficiently using $\textsc{Select}_1$ queries. A $\textsc{Select}_1(i)$ query over A_{high} returns the position of the i-th one, for $1 \leq i \leq n$. It follows that $x_h = \textsc{Select}_1(i) - i$.

The extra bits used for $\textsc{Select}_1$ as well as the number of cache misses claimed in Point 1. of Theorem 1 follow by using Theorem 2 for A_{high} ($u < 3n$). We explain Theorem 2 below.

Select queries. Clark [1997] shows that $\textsc{Select}_1$ can be supported in $O(1)$ time but the data structure requires a non-trivial space usage in practice and many distinct memory accesses, resulting in several cache misses.

We describe the solution by Okanohara and Sadakane [2007], the *DArray* index, which is inspired by Clark's solution and we use in practice. (We assume $\textsc{Select}_1$ queries throughout the presentation although one can obviously flip the ones into zeros to support $\textsc{Select}_0$ as well.)

Theorem 2 (DArray) Consider a bitvector of u bits. There exists an index that takes $o(u)$ bits and supports $\textsc{Select}_1$ queries in at most $2 + C(O(\log^4 u))$ cache misses.

Let L, L_2, and L_3 be integer quantities to be fixed later. Let z be the number of ones in the bitvector. The bitvector is split into variable-length blocks, each containing L ones (except for, possibly, the last block). A block is called *sparse* if its length is larger than L_2, *dense* otherwise. Sparse blocks are represented verbatim, i.e., the positions of the L ones are coded using $\log_2(u)$-bit integers. A dense block, instead, is sparsified: we keep one 1-bit position every L_3 such positions. The positions are coded relatively to the beginning of each block, hence taking $\log_2(L_2)$ bits per position. The data structure therefore stores three arrays, I, S, D. The *inventory* array $I[1..z/L]$ is such that $I[i] := \textsc{Select}_1((i-1)L + 1)$ if block i is dense; otherwise, $I[i] = -p - 1$ where p is the start position in S of the 1-bit

© The Author 2025. Published by Oxford University Press. All rights reserved. For permissions, please e-mail: journals.permissions@oup.com

positions of block i. The space for I is therefore $z/L \cdot \log_2(u)$ bits. The array S holds the positions of the L ones in sparse blocks. As we have at most u/L_2 sparse blocks, its space is $u/L_2 \cdot L \cdot \log_2(u)$ bits at most. Lastly, the array $D[1..z/L_3]$ is such that $D[i] := \text{SELECT}_1((i-1)L_3+1) - I[j]$ if block $j = \lceil \frac{(i-1)L_3+1}{L} \rceil$ is dense, or $D[i] := -1$ otherwise. Its space is $z/L_3 \cdot \log_2(L_2)$ bits.

A $\text{SELECT}_1(i)$ query, $1 \le i \le z$, first checks $p = I[\lceil i/L \rceil]$: if $p < 0$, then the block is sparse and the query is answered as $S[-p-1+((i-1) \bmod L)]$; otherwise the position $D[\lceil i/L_3 \rceil]$ is retrieved and a sequential scan of at most L_2 bits is executed starting from position $p + D[\lceil i/L_3 \rceil]$. It follows that the number of cache misses per query is: 2, if i belongs to a sparse block; $2 + C(L_2)$, if i belongs to a dense block.

Choosing $L = O(\log^2 u)$, $L_2 = O(\log^4 u)$, and $L_3 = O(\log u)$, all the three arrays I, S, and D together take $o(u)$ bits and the number of cache misses per query is at most $C_{\text{sel}} = 2 + C(O(\log^4 u))$. (In practice, our implementation of the Darray uses $L = 2^{10}$, $L_2 = 2^{16}$, and $L_3 = 2^5$.)

Successor. Using SELECT_0 queries on A_{high}, it is also possible to support the query $\text{SUCCESSOR}(x)$. From x, we compute $x_h = \lfloor x/2^\ell \rfloor$ and $i = p - x_h$ with $p = \text{SELECT}_0(x_h)$. For $x_h > 0$, this indicates that there are i values whose high parts are *less* than x_h (when $x_h = 0$, we let $i = 0$). On the other hand, $j = \text{SELECT}_0(x_h+1) - x_h$ gives us the position of the first element having high bits larger than x_h. Since a cluster contains at most $2^\ell \le U/n$ elements, we have that $j - i \le 2^\ell \le U/n$ elements, and the successor could be determined by binary searching in the range $A[i..j]$ for a total of $O(\log(U/n)(1 + C_{\text{sel}}))$ cache misses. This algorithm is not, however, cache-efficient. It is better in practice to answer the query by scanning A from the $(i+1)$-th element. We follow this latter approach as it matches our implementation. (It relies on the fact that Δ_A is small for practical applications of Elias-Fano, like SSHash, albeit $\Delta_A = O(n)$ in the worst case.) When scanning from the $(i+1)$-th element, the following cases can happen:

1. The bit in position $p+1$ of A_{high} is 0: then cluster x_h is empty and the successor of x is $A[i+1]$ (minimum element in the next non-empty cluster). The low bits of $A[i+1]$ are retrieved with 1 cache miss, whereas the high bits are computed by scanning A_{high} from position $p+1$ until the next bit set. Since the longest run of zeros between two consecutive ones in A_{high} has length Δ_A, $C(\Delta_A)$ cache misses are issued during the scan.
2. The bit in position $p+1$ of A_{high} is 1, so the cluster is not empty. The elements in the cluster all have the same high bits x_h. Now, two cases can happen:
   a. The successor is not larger than the largest element in the cluster, so it belongs to the cluster. Scanning up to U/n elements therefore costs $C(U/n) + C(\ell \cdot U/n)$ cache misses.
   b. The successor is larger than the largest element in the cluster, so it is the minimum in the next non-empty cluster. The cost is at most $C(U/n) + C(\ell \cdot (U/n+1)) + C(\Delta_A)$.

Using again Theorem 2 on the zeros of A_{high} to implement SELECT_0, the extra bits and number of cache misses claimed in Point 2. of Theorem 1 follow.

Lastly, Point 3. of Theorem 1 illustrates a more space-consuming alternative that, on the other hand, supports faster SUCCESSOR. The idea is to use an extra array $hints[1..\lfloor U/2^\ell \rfloor]$ such that $hints[i] = \text{SELECT}_0(i)$, for $i = 1..\lfloor U/2^\ell \rfloor$. As $n \le \lfloor U/2^\ell \rfloor < 2n$ and $|A_{\text{high}}| < 3n$, the space bound follows. Instead of computing $\text{SELECT}_0(x_h)$, this value is readily available as $hints[x_h]$ (in the general case when $x_h > 0$).

Table 1. Number of cache misses: theory and practice. These results are for $k = 31$ and SSHash regular, for random positive LOOKUP queries.

(a) Theorem 2

	Cod	Human	HPRC
Case 1, theory	43.9	58.1	149.9
Case 1, practice	32.8	49.2	136.2
Case 2, theory	20.0	23.5	23.5
Case 2, practice	16.1	22.5	21.5

(b) Theorem 3

	Cod	Human	HPRC
Case 1, theory	7	7	7
Case 1, practice	7.05	6.1	6.8
Case 2, theory	152.2	152.9	135.5
Case 2, practice	143.6	147.1	132.2
Case 3, theory	10	10	10
Case 3, practice	10.5	8.7	10

2. Cache miss analysis: theory and practice

As discussed in Section 2 of the main paper, we model the number of cache misses involved during a read of Q bits from main memory to the cache with $C(Q) := \lceil Q/B \rceil$, where B is the cache line size. In this section we validate this model and show that it is accurate under proper tuning. In particular, we compare the number of theoretical cache misses of LOOKUP claimed in Theorem 2 and Theorem 3 of the main paper with the *actual* number of cache misses measured using the Linux `perf` tool (command: `perf stat -B -e cache-misses`).

For ease of presentation, we report again below the number of theoretical cache misses from Theorem 2 and Theorem 3 of the main paper. Both are valid for a $\text{LOOKUP}(x)$ query, with $z = |\text{loc}(\text{MINI}(x))|$.

Theorem 2: previous SSHash. The number of cache misses is at most

1. $1 + C_{\text{acc}} + C(z\log_2(N)) + z(C_{\text{succ}} + C(4k-2m))$ if $1 \le z \le 2^l$;
2. $4 + C_{\text{acc}} + C_{\text{succ}} + C(4k-2m)$ otherwise.

Theorem 3: current SSHash. The number of cache misses is at most

1. $2 + C'_{\text{succ}} + C(2k)$ if $z = 1$;
2. $3 + C(z\log_2(N)) + z(C'_{\text{succ}} + C(2k))$ if $2 \le z \le 2^l$;
3. $5 + C'_{\text{succ}} + C(2k)$ otherwise.

The values of C_{acc}, C_{succ}, C'_{succ}, and C_{scan} are:

- $C_{\text{acc}} = 3 + C(O(\log^4 M))$,
- $C_{\text{succ}} = 2 + C(O(\log^4 |\mathcal{S}|)) + C_{\text{scan}}$,
- $C'_{\text{succ}} = 1 + C_{\text{scan}}$,
- $C_{\text{scan}} = C(N/|\mathcal{S}|) + C((N/|\mathcal{S}|+1)\log_2(N/|\mathcal{S}|)) + C(\Delta_P)$.

Fixing the parameters and result. We fix $B = 512$, which is a very common cache line size and, indeed, that of our testing machine. For the choice of k and m in our experimental analysis (Section 8 of the main paper), we have $C(2k) = C(4k - 2m) = 1$ for $k = 31$ and $m < k$. Although the longest run of zeros on the high bitvector of the Elias-Fano can can be as large as $O(n)$ in the worst case, Δ_P is actually small on tested datasets. For example, it is 140 on the whole human genome. So, we let $C_{\text{scan}} = 3$. We let $C(O(\log^4 M)) = 6 \cdot C(O(\log^4 |\mathcal{S}|))$ for Theorem 2 because, in practice, the *sizes* array is approximately 6 times larger than the P array. We use $C(O(\log^4 |\mathcal{S}|)) = 1$ for Cod but $C(O(\log^4 |\mathcal{S}|)) = 1.5$ for both Human and HPRC as their respective indexes are much larger than that for Cod. For the value of z we use the average number of positions j inspected by 10^6 random positive Lookup queries. Lastly, we have $\log_2(N)$ equal to 30, 32, and 34 for Cod, Human, and HPRC respectively.

Table 1 reports the result of the comparison: for every case and dataset, the model closely matches the actual number of cache misses.

3. Construction

We describe a multi-threaded construction algorithm for the new layout of SSHash described in Section 6 of the main paper, designed to scale to large collections using external memory and a fixed RAM budget. The construction takes as input a compressed collection of strings (in FASTA format and compressed, for example with `gzip`) and proceeds as a pipeline of streaming and sorting phases. In short: minimizers are first generated by streaming through the strings, in parallel; then, sorted in external memory; and finally laid out contiguously. The construction steps are as follows.

1. Input encoding. Each string in the input is decompressed incrementally, 2-bit encoded using SIMD instructions, and concatenated in S.

2. Parallel minimizer computation. The obtained string S is split into chunks, one chunk per thread. The RAM dedicated to the construction is split evenly among threads. Each thread computes the minimizers in its chunk in a *streaming* fashion. We use the folklore *re-scan* method which performs better in practice than the monotone-queue approach we used before (see the discussion in [Zheng et al., 2025]). For each minimizer occurrence, the thread emits the tuple (ϕ, j, p, v) into a thread-local in-memory buffer. The tuple comprises: the minimizer itself ϕ (as a 2-bit encoded string), its occurrence j in S, the offset p indicating that the super-k-mer of minimizer ϕ starts in S at position $j - p + 1$, and lastly v, the number of k-mers in its super-k-mer. (These last two quantities, p and v, are used to build efficiently the skew index in the last step.) When the buffer reaches its dedicated capacity, the tuples in the buffer are sorted by the components (ϕ, j) and the buffer is flushed to disk as a sorted run.

3. External merge using a winner tree. The sorted runs on disk are merged into a single run using a classic multi-way external merge algorithm. However, instead of a min-heap, a *winner tree* is used to select the minimum element at each step of the merge. A winner tree is a complete binary-tree, like a min-heap, but it performs only one comparison per tree level when updating the minimum (compared to two, as spent by a min-heap), yielding a ≈30% speedup in our experiments. Knuth [1998] (Section 5.4.1) gives a description of tournament trees.

Table 2. Distinct k-mers and minimizer lengths (m) for SSHash.

Collection	$k = 31$; m	$k = 63$; m
Cod	502,465,200; 20	556,585,658; 24
Kestrel	1,150,399,205; 20	1,155,250,667; 24
Human	2,505,678,680; 21	2,771,316,093; 25
NCBI-v	376,205,185; 19	412,515,880; 23
SE	894,310,084; 21	1,524,904,156; 31
HPRC	3,718,120,949; 21	5,926,785,469; 31

4. MPHF construction. From the merged minimizer stream on disk, we build the MPHF f using external memory and multiple threads. Our implementation uses PTHash as choice of MPHF [Pibiri and Trani, 2021, 2023].

5. Resorting tuples in MPHF order. The minimizer tuples on disk are sorted again according to the identifier assigned to minimizers by f. As a result, all occurrences of the same minimizer become contiguous in a file on disk. This process is implemented, again, with a parallel external-memory merge sort.

6. Locate sets construction. Since minimizer tuples are now laid out consecutively on disk, the arrays T, G, L, and H are all computed by scanning the tuples sequentially.

7. Skew index construction. During the scan, minimizers occurring more than 2^l times are detected and the $r - l + 1$ partitions are built one after the other. Consider a tuple (ϕ, j, p, v) such that $2^i < |\text{loc}(\phi)| \le 2^{i+1}$ for some $i \ge l$. All the k-mers $x \in S[j - p - 1..j - p + v + k)$ are added to the set K_i under formation. As soon as the next processed minimizer has a locate set larger than 2^{i+1}, the MPHF f_i is built (in parallel) for K_i, V_i laid out consequently, and the process continues with the next partition.

4. Parameter sweep

The performance of SSHash is dependent on the selection of its structural parameters. The space-time tradeoff is governed by two main parameters: the minimizer length m and the skew index threshold l.

- **Minimizer length, m.** This parameter dictates the fundamental space-time tradeoff of the index. A smaller m optimizes index space but results in larger locate sets (since more k-mers share the same minimizer), which in turn makes query resolution slower. Conversely, a larger m makes queries significantly faster by reducing the size of the locate sets, but consumes more space due to indexing a larger number of distinct minimizers.
- **Skew index threshold, l.** This parameter handles highly abundant minimizers. Any k-mer whose minimizer appears more than 2^l times is routed to the skew index. Choosing a small l accelerates queries because a larger fraction of difficult, high-frequency k-mers are solved quickly via the skew index. However, populating the skew index more heavily naturally consumes more memory space.

Varying the minimizer length. Based on the m-sweep benchmark plots in Figure 1, the impact of the minimizer length across the Human and SE datasets is highly visible. For $k = 31$ (represented by circles), we observe a distinct downward-sloping curve. As m increases from 17 to 25 (or up to 31 for SE), the lookup time drops drastically while the index space grows steadily. For $k = 63$ (squares), increasing m yields

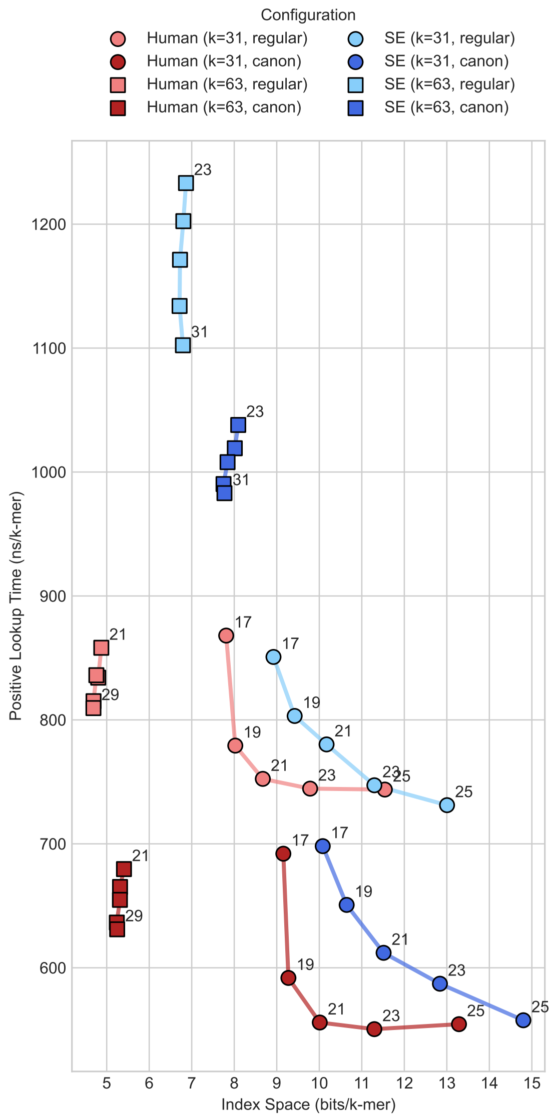

Fig. 1. Space-time tradeoff of SSHash when varying the minimizer length m across Human and SE datasets.

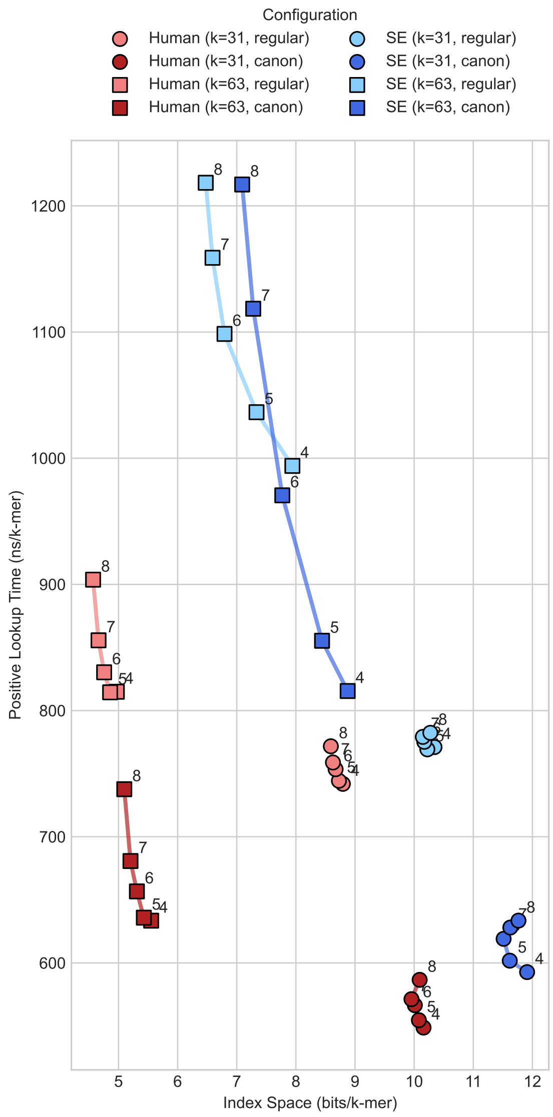

Fig. 2. Space-time tradeoff of SSHash when varying the skew index threshold l across Human and SE datasets.

a steep drop in query time with only a marginal penalty in space, making larger m values highly attractive.

Varying the skew index threshold. The l-sweep plots in Figure 2 isolate the effect of the skew index threshold, revealing how managing high-frequency minimizers impacts performance. As expected, when l decreases from 8 down to 4, there is a clear visual migration down and to the right for every single configuration. Query time improves as more k-mers are offloaded to the fast skew index, at the direct cost of increased bits/k-mer. For some configurations however, notably the Human dataset at $k = 31$ (canonical), dropping l from 5 to 4 yields almost no improvement in query time but still incurs a space penalty. This indicates that a good threshold is $l = 5$ or $l = 6$ for this specific case. The SE dataset with $k = 63$ (regular and canonical) shows a massive vertical spread. For these configurations, relying more heavily on the skew index (smaller l) is highly effective at driving down query times from over 1200 to under 1000 ns/k-mer.

5. Streaming queries

Figure 3 shows the performance for streaming Lookup queries on three different scenarios: "high-hit" (most k-mers are found in the indexes), "low-hit" (most k-mers are *not* found), and a "mixed" workload where the previous query workloads are mixed 50-50%. As explained in Section 8 of the main paper, the indexes stream through FASTQ reads and each read set contains several million reads. All reads are available at https://zenodo.org/records/17582116 for reproducibility.

High-hit workloads are relevant to assess the capability of the indexes to take advantage of consecutive query k-mers, whereas low-hit workloads mostly test the ability to reject alien k-mers. Since both cases are relevant in practice, the mixed-hit workload alternates between the two cases.

Overall, all evaluated indexes exhibit robust query times, delivering consistent and predictable performance across different workloads without significant variation. Across all benchmarks, SSHash offers the fastest query time though, being

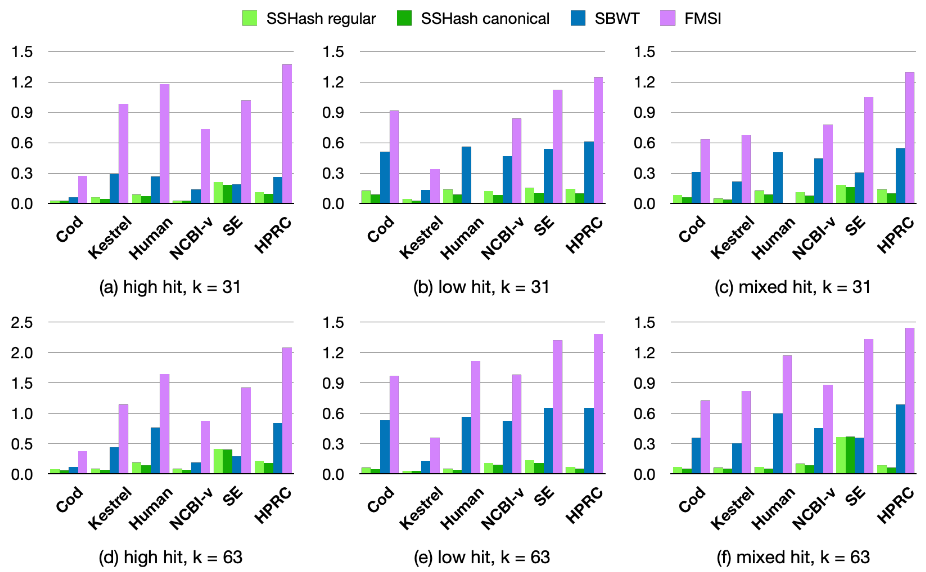

Fig. 3. Streaming query efficiency in avg. μs/k-mer. (In plot (b), FMSI is not reported for the Human dataset because the corresponding implementation generated a segmentation fault.)

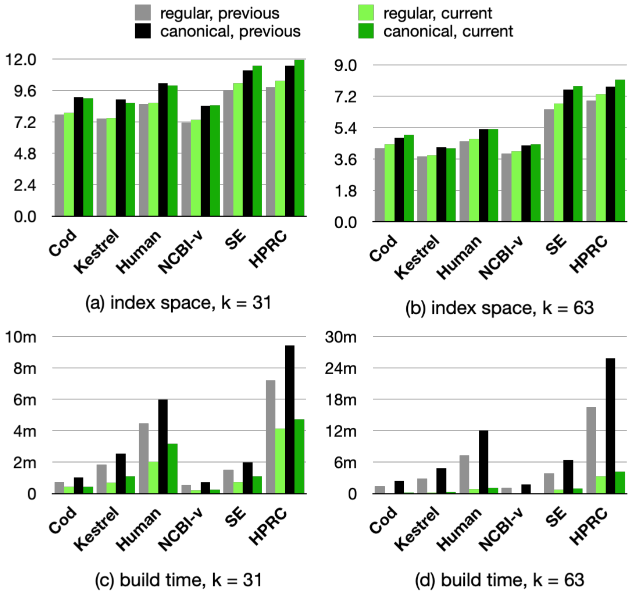

Fig. 4. Comparison between previous and current SSHash: index space (in avg. bits/k-mer) and build time.

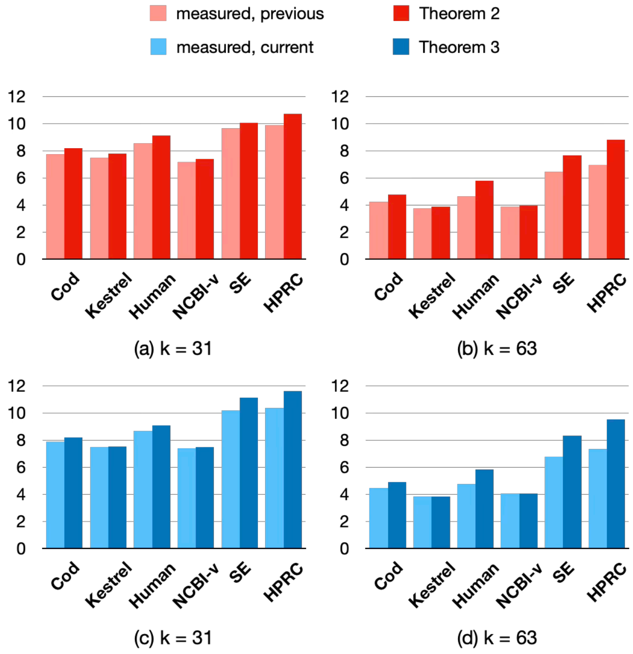

Fig. 5. Comparison between measured index space and that from Theorem 2 and Theorem 3 of the main paper. Space is reported in avg. bits/k-mer. The constants in the asymptotic terms $\Theta(\alpha)$ and $\Theta(M)$ are the same from both theorems and equal to 2.5 and 3.0 respectively, which are faithful to our implementation.

several times faster than its competitors. The only exception is the SE datasets (high and mixed workloads), where the SBWT gives competitive runtimes.

6. Experimental comparison against the previous version

We compare the version of SSHash from this work (referred to as *current* in the plots) and the previous version[1], using the same datasets (see Table 2), machine, and methodology described in Section 8 of the main paper. In general, the current version outperforms the previous one under every aspect and consistently on all tested dataset.

Figure 4 illustrates the space and build time of the two versions. The space is very similar between the two versions. Furthermore, Figure 5 shows the comparison between the actual, measured, space and the bounds from Theorem 2 and Theorem 3 in the main paper. In both cases, the bounds are

[1] GitHub commit: `a2a2d26817fe3f476ceac44809c333ede6622ff3`.

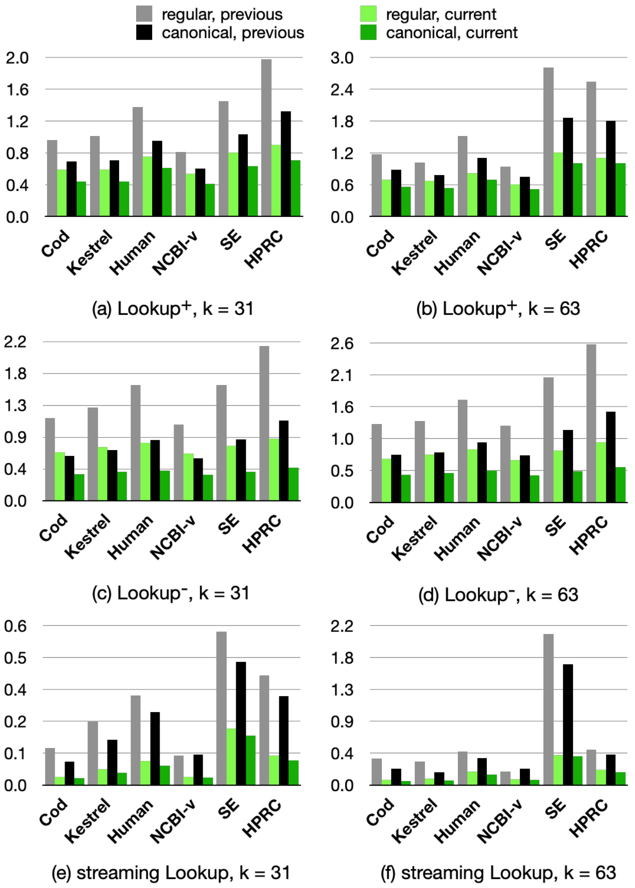

Fig. 6. Comparison between previous and current SSHash: query times are reported in avg. μs/k-mer.

quite close to the measured space and most of the difference comes from overestimating the cost of the skew index with $\alpha(\log_2(N) + \Theta(1))$ bits.

The current version is between $2 - 3\times$ faster to build, on average for $k = 31$. This result improves for larger k; for example, it is up to $6\times$ faster for $k = 63$. The better build time is due to better multi-threading, faster minimizer computations over streams, and faster merging in external memory which the previous version supported only partially.

Figure 6, instead, shows the query times of the two versions. (Query time for Access is almost the same between the two versions, as apparent from the tables in this document, so we do not discuss it in the following.) Avoiding the scan of super-k-mers and the cache-efficient layout both contribute to faster random Lookup queries. The simpler logic of the streaming Lookup algorithm paired with the more efficient random Lookup, results in $2 - 3\times$ faster streaming queries. In particular, the refined logic consistently increases the extension rate compared to the previous version, of about 15% for $k = 31$ (Figure 7).

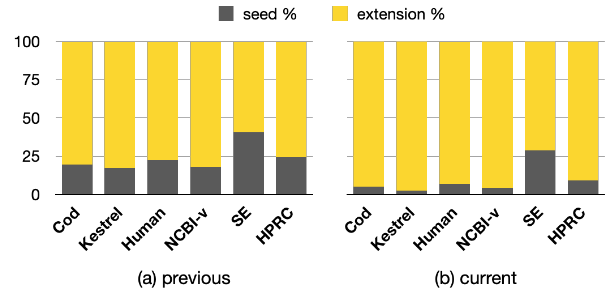

Fig. 7. Seed vs. extension rate for streaming Lookup queries, for $k = 31$.

References

D. Clark. *Compact pat trees.* PhD thesis, University of Waterloo, 1997.

P. Elias. Efficient storage and retrieval by content and address of static files. *Journal of the ACM*, 21(2):246–260, 1974.

R. M. Fano. On the number of bits required to implement an associative memory. *Memorandum 61, Computer Structures Group, MIT*, 1971.

D. E. Knuth. *The Art of Computer Programming: Sorting and Searching, Volume 3.* 1998.

D. Okanohara and K. Sadakane. Practical entropy-compressed rank/select dictionary. In *ALENEX*, pages 60–70, 2007.

G. E. Pibiri and R. Trani. PTHash: Revisiting FCH minimal perfect hashing. In *SIGIR*, pages 1339–1348, 2021.

G. E. Pibiri and R. Trani. Parallel and external-memory construction of minimal perfect hash functions with PTHash. *Transactions on Knowledge and Data Engineering*, 36(3):1249–1259, 2023.

A. Zheng, I. Lee, V. S. Shivakumar, O. Y. Ahmed, and B. Langmead. Fast and flexible minimizer digestion with digest. *Bioinformatics*, 41(7), 2025.

Table 3. Index space and construction efficiency for current SSHash.

(a) regular

k	Collection	bits/k-mer	GB	build time
31	Cod	7.89	0.50	26s
	Kestrel	7.50	1.08	43s
	Human	8.67	2.72	2m 1s
	NCBI-v	7.37	0.35	13s
	SE	10.17	1.14	45s
	HPRC	10.35	4.81	4m 8s
63	Cod	4.44	0.31	12s
	Kestrel	3.82	0.55	16s
	Human	4.76	1.65	54s
	NCBI-v	4.05	0.21	5s
	SE	6.79	1.29	44s
	HPRC	7.33	5.43	3m 20s

(b) canonical

k	Collection	bits/k-mer	GB	build time
31	Cod	9.01	0.57	26s
	Kestrel	8.67	1.25	1m 6s
	Human	10.01	3.14	3m 10s
	NCBI-v	8.48	0.40	16s
	SE	11.51	1.29	1m 6s
	HPRC	11.93	5.54	4m 45s
63	Cod	4.97	0.35	15s
	Kestrel	4.22	0.61	19s
	Human	5.31	1.84	1m 9s
	NCBI-v	4.46	0.23	7s
	SE	7.77	1.48	58s
	HPRC	8.14	6.03	4m 13s

Table 4. Query efficiency for current SSHash. Timings for Lookup and Access are in avg. microseconds per k-mer. For Streaming (high-hit), we report avg. nanoseconds per k-mer.

(a) regular

k	Collection	Lookup$^+$	Lookup$^-$	Access	Streaming
31	Cod	0.59	0.67	0.28	30
	Kestrel	0.59	0.74	0.28	60
	Human	0.75	0.80	0.36	90
	NCBI-v	0.54	0.65	0.26	30
	SE	0.80	0.76	0.36	213
	HPRC	0.90	0.86	0.54	112
63	Cod	0.69	0.71	0.29	77
	Kestrel	0.67	0.78	0.33	86
	Human	0.82	0.86	0.36	188
	NCBI-v	0.61	0.69	0.28	85
	SE	1.20	0.85	0.41	412
	HPRC	1.10	0.98	0.64	213

(b) canonical

k	Collection	Lookup$^+$	Lookup$^-$	Access	Streaming
31	Cod	0.44	0.37	0.28	26
	Kestrel	0.44	0.40	0.28	46
	Human	0.61	0.42	0.35	74
	NCBI-v	0.41	0.36	0.26	29
	SE	0.63	0.40	0.36	186
	HPRC	0.71	0.46	0.54	93
63	Cod	0.56	0.45	0.29	60
	Kestrel	0.54	0.48	0.33	66
	Human	0.69	0.52	0.36	146
	NCBI-v	0.52	0.44	0.28	72
	SE	1.00	0.51	0.41	400
	HPRC	1.00	0.58	0.64	181

Table 5. Index space and construction efficiency for previous SSHash.

(a) regular

k	Collection	bits/k-mer	GB	build time
31	Cod	7.75	0.49	43s
	Kestrel	7.47	1.07	1m 50s
	Human	8.56	2.68	4m 27s
	NCBI-v	7.17	0.34	33s
	SE	9.65	1.08	1m 30s
	HPRC	9.88	4.59	7m 14s
63	Cod	4.23	0.29	1m 28s
	Kestrel	3.76	0.54	2m 56s
	Human	4.63	1.60	7m 20s
	NCBI-v	3.90	0.20	1m 4s
	SE	6.46	1.23	3m 51s
	HPRC	6.94	5.14	16m 32s

(b) canonical

k	Collection	bits/k-mer	GB	build time
31	Cod	9.11	0.57	1m 1s
	Kestrel	8.93	1.28	2m 34s
	Human	10.15	3.18	6m
	NCBI-v	8.44	0.40	44s
	SE	11.15	1.25	1m 59s
	HPRC	11.50	5.35	9m 26s
63	Cod	4.81	0.33	2m 22s
	Kestrel	4.28	0.62	4m 55s
	Human	5.30	1.83	12m 5s
	NCBI-v	4.37	0.23	1m 45s
	SE	7.59	1.45	6m 25s
	HPRC	7.76	5.75	25m 53s

Table 6. Query efficiency for previous SSHash. Timings for Lookup and Access are in avg. microseconds per k-mer. For Streaming (high-hit), we report avg. nanoseconds per k-mer.

(a) regular

k	Collection	Lookup$^+$	Lookup$^-$	Access	Streaming
31	Cod	0.96	1.14	0.29	140
	Kestrel	1.00	1.29	0.26	239
	Human	1.37	1.60	0.37	337
	NCBI-v	0.81	1.05	0.29	111
	SE	1.45	1.60	0.39	578
	HPRC	1.97	2.14	0.60	412
63	Cod	1.18	1.30	0.31	363
	Kestrel	1.00	1.40	0.27	329
	Human	1.55	1.70	0.36	461
	NCBI-v	0.94	1.27	0.30	184
	SE	2.81	2.04	0.44	2084
	HPRC	2.54	2.58	0.68	488

(b) canonical

k	Collection	Lookup$^+$	Lookup$^-$	Access	Streaming
31	Cod	0.69	0.62	0.29	89
	Kestrel	0.71	0.70	0.27	172
	Human	0.95	0.84	0.37	276
	NCBI-v	0.60	0.59	0.28	117
	SE	1.03	0.85	0.39	465
	HPRC	1.32	1.11	0.60	335
63	Cod	0.88	0.78	0.31	228
	Kestrel	0.78	0.82	0.27	180
	Human	1.11	0.98	0.36	371
	NCBI-v	0.75	0.77	0.30	229
	SE	1.86	1.18	0.44	1671
	HPRC	1.81	1.48	0.68	425
